# Genome assembly and isoform analysis of a highly heterozygous New Zealand fisheries species, the tarakihi (*Nemadactylus macropterus*)

**DOI:** 10.1093/g3journal/jkac315

**Published:** 2022-12-08

**Authors:** Yvan Papa, Maren Wellenreuther, Mark A Morrison, Peter A Ritchie

**Affiliations:** School of Biological Sciences, Victoria University of Wellington, Wellington 6012, New Zealand; Seafood Production Group, The New Zealand Institute for Plant and Food Research Limited, Nelson 7010, New Zealand; School of Biological Sciences, The University of Auckland, Auckland 1010, New Zealand; National Institute of Water and Atmospheric Research, Auckland 1010, New Zealand; School of Biological Sciences, Victoria University of Wellington, Wellington 6012, New Zealand

**Keywords:** fish, genomics, Iso-Seq, marine, teleost, transcriptome

## Abstract

Although being some of the most valuable and heavily exploited wild organisms, few fisheries species have been studied at the whole-genome level. This is especially the case in New Zealand, where genomics resources are urgently needed to assist fisheries management. Here, we generated 55 Gb of short Illumina reads (92× coverage) and 73 Gb of long Nanopore reads (122×) to produce the first genome assembly of the marine teleost tarakihi [*Nemadactylus macropterus* (Forster, 1801)], a highly valuable fisheries species in New Zealand. An additional 300 Mb of Iso-Seq reads were obtained to assist in gene annotation. The final genome assembly was 568 Mb long with an N50 of 3.37 Mb. The genome completeness was high, with 97.8% of complete Actinopterygii Benchmarking Universal Single-Copy Orthologs. Heterozygosity values estimated through *k*-mer counting (1.00%) and bi-allelic SNPs (0.64%) were high compared with the same values reported for other fishes. Iso-Seq analysis recovered 91,313 unique transcripts from 15,515 genes (mean ratio of 5.89 transcripts per gene), and the most common alternative splicing event was intron retention. This highly contiguous genome assembly and the isoform-resolved transcriptome will provide a useful resource to assist the study of population genomics and comparative eco-evolutionary studies in teleosts and related organisms.

## Introduction

The tarakihi or jackass morwong (*Nemadactylus macropterus*, Centrarchiformes: Cirrhitioidei, NCBI Taxon ID: 76931) is a species of demersal marine teleost fish that is widely distributed around all inshore areas of New Zealand and along the southern coasts of Australia. It is distinguishable from other New Zealand “morwongs” by the black saddle across its nape (Roberts *et al*., 2015) and displays a single elongated pectoral fin ray that is characteristic of *Nemadactylus* species (Ludt *et al*., 2019). The species and its genus have been recently moved from the Cheilodactylidae to the Latridae following extensive revision of the taxonomy of both families, which until then was poorly understood (Kimura *et al*., 2018; Ludt *et al*., 2019). Tarakihi is an important commercial and recreational inshore fishery, especially in New Zealand, where more than 5,000 tonnes are harvested every year (Fisheries New Zealand, 2018). Like many other fisheries species, tarakihi stocks have been heavily fished over the past century. As a result, the spawning biomass is now concerningly depleted to numbers below the fisheries management soft limit of 20% on the east coast of New Zealand, where fishing effort is highest (Langley, 2018). Low effective population size and spawning biomass are of concern for the long-term sustainability of this species, particularly with added and increasing environmental pressures due to global warming. Climate change is already having an impact on marine ecosystems and is expected to affect the distribution and productivity of many fisheries species (Burrows *et al*., 2011; Ramos *et al*., 2018; Babcock *et al*., 2019).

The application of genome-wide markers for tarakihi fisheries management has been limited by the lack of a reference genome. Consequently, the first step in developing new genomic resources for this species is to assemble a high-quality reference genome that can be used to develop high-resolution markers for determining the genetic stock structure. This will offer the potential to estimate gene flow levels and detect adaptive genetic variation (Papa, Morrison, *et al*., 2022). Incorporating adaptive genetic variation, along with neutral variation, will greatly improve how the genetic data can be used for fisheries management (Bernatchez *et al*., 2017; Benestan, 2019; Papa, Oosting, *et al*., 2021; Thomson *et al*., 2021).

Genome assembly quality, contiguity, and completeness depend on the available technology used. While short-read Illumina sequencing produces highly accurate reads, their short length (<200 bp) makes them problematic for the assembly of highly repetitive segments of the genome. Complex genomes often result in highly fragmented assemblies (Koren *et al*., 2012; Rice & Green, 2019). Combining short reads with less accurate long-read sequencing technologies leads to more contiguous genome assemblies (Austin *et al*., 2017; Zimin, Puiu, *et al*., 2017; Zimin, Stevens, *et al*., 2017; Tan *et al*., 2018; Dhar *et al*., 2019; Jiang *et al*., 2019; Wiley & Miller, 2020). On the other hand, the relatively young circular consensus sequencing (CCS) PacBio technology produces reads that are both thousands of bp long and highly accurate. CCS long-read DNA sequencing can be applied to DNA (HiFi reads) and RNA (i.e. isoform sequencing or Iso-Seq). Iso-Seq allows for the sequencing of complete, uninterrupted mRNAs, which enables the accurate characterization of isoforms (An *et al*., 2018; Byrne *et al*., 2019; Gao *et al*., 2019; Hoang & Henry, 2021). Iso-seq has been used to annotate de novo genome assemblies of nonmodel organisms like the cave nectar bat (*Eonycteris spelaea*) (Wen *et al*., 2018), the pharaoh ant (*Monomorium pharaonis*) (Gao *et al*., 2020), the red-eared slider turtle (*Trachemys scripta elegans*) (Simison *et al*., 2020), and the sponge gourd (*Luffa* spp.) (Pootakham *et al*., 2020), allowing for the characterization of both gene functions and alternative splicing (AS) patterns in these species.

The main goal of this study was to complete the first tarakihi genome assembly. This was achieved by using a combination of short-read Illumina and long-read Nanopore sequencing data. Four assembly pipelines were compared, three of which used algorithms implemented in MaSuRCA for hybrid assembly, and a fourth pipeline was based on a trial run of low-coverage DNA sequence reads (4 Gb) generated using the PacBio HiFi platform. Iso-Seq data were used to assist with gene annotation and the identification of gene isoforms.

## Materials and methods

### Tissue collection and nucleotide extraction

**Fig. 1.**
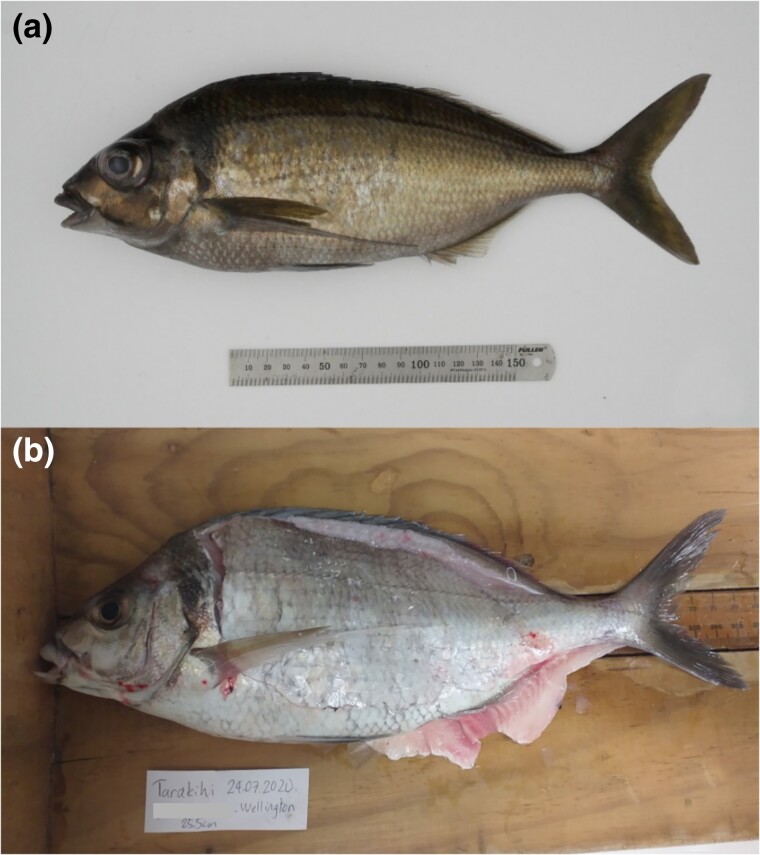
Tarakihi specimens used in this study. a) TARdn1: captive-bred specimen used for Illumina and Nanopore sequencing and b) TARdn2: wild-caught specimen used for HiFi sequencing and Iso-Seq.

Tissues for Illumina and Nanopore sequencing were collected from a freshly vouchered *N. macropterus* specimen (male, standard length: 285 mm, weight: 460 g). The specimen was a captive-bred from Plant & Food Research, Nelson, New Zealand (Fig. 1a) and is thereby referred to as TARdn1 (for “tarakihi de novo”). A caudal fin clip and a heart piece were stored in 96% EtOH, and a kidney piece was stored in DESS (20% DMSO, 0.25 M EDTA, NaCl saturated solution). Total genomic DNA was extracted from these tissues using a high-salt extraction protocol adapted from that of Aljanabi & Martinez (1997), which included an RNase treatment, and then suspended in Tris-EDTA buffer (10 mM Tris–HCl pH 8.0 and 1 mM EDTA). The integrity of DNA fragments was assessed by gel electrophoresis in 1% agarose. The purity and quantity of DNA (concentration >200 ng/µl, A260/280≈1.8, A60/230≈2, total weight >20 µg) were estimated with CLARIOstar spectrometer (BMG Labtech). Purified DNA samples were sent to Annoroad Gene Technology Co. Ltd (Beijing, China) and NextOmics Biosciences Co. Ltd (Wuhan, China) for Illumina and Nanopore library preparation and sequencing.

Tissues for HiFi sequencing and Iso-Seq were obtained from a wild specimen (male, standard length: 255 mm) captured by a recreational fisherman at Kau Bay, in Wellington Harbour (New Zealand), thereby referred to as TARdn2 (Fig. 1b). Tissues were collected c. 6 h after capture and flash-frozen in liquid nitrogen. Five pieces of tissues were sent to BGI Tech Solutions Co. Ltd (Hong Kong, China): one tissue (heart, weight: 2 g) for DNA extraction and HiFi sequencing and four tissues (liver, white muscle, brain, and spleen, weight: c. 150 mg) for RNA extraction and Iso-Seq. DNA and RNA were extracted by BGI using a phenol-chloroform method.

### Library preparations and sequencing

Before sequencing, the genome size of *N. macropterus* was estimated to be about 700 Mb (see *Methods* in Supplementary File 1). The quantity of Illumina and Nanopore bases to be sequenced was tuned for a deep 85× Illumina coverage (c. 60 Gb) and 140× Nanopore coverage (c. 100 Gb), following sequencing provider recommendations. For Illumina reads, DNA samples were sheared for a fragment insert size of 350 ± 50 bp. Approximately 200 million of 150 bases pair-end reads were generated using the HiSeq X System (Illumina). Raw Illumina reads were filtered for adapter contamination, base uncertainty, and Quality Value. For Nanopore sequencing, a DNA library of 20–40 Kb fragments was loaded into two flow cells on PromethION (Oxford Nanopore Technologies). The HiFi library was prepped with SMRTbell Express Template Prep Kit 2.0 (Pacific Biosciences) and CCS was performed on one-third of an SMRT Cell 8 M with a PacBio Sequel II sequencer. Four Iso-Seq libraries of 0–5 Kb insert sizes (one per tissue) were generated using the SMRTbell Express Template Prep Kit 2.0. The multiplexed libraries were sequenced on one SMRT Cell 8 M with a PacBio Sequel II sequencer, resulting in 3.6 million polymerase reads from which subreads were extracted (see *Methods* in Supplementary File 1 for more details on library preparation, sequencing, and preliminary filtering).

### Quality, contamination, and mitochondrial filtering

Primary quality filtering resulted in 405.2 million Illumina pair-end reads (60.78 Gb). Quality metrics of these filtered reads were visualized with FastQC v0.11.7 (Andrews, 2018) before proceeding to the next steps. Kraken v2.0.7-beta (Wood *et al*., 2019) was used to detect and filter contamination from archaeal, bacterial, viral, and human sequences based on the MiniKraken2 v2 8GB database (Wood, 2019). The 9.25% of reads that were classified as contaminants were discarded, leading to 367.8 million noncontaminated reads (55.16 Gb) (Table 1). The mitogenome sequence (16,650 bp) was then retrieved and discarded from the genome assembly (see details in *Methods* in Supplementary File 1). A total of 99.18 Gb was obtained from the raw unfiltered Nanopore reads, with an average read length above 6 Kb and a maximum length above 1 Mb (Table 1). Nanopore reads were filtered and trimmed for minimum length (500 bp) and a minimum average read quality score of 7 (c. 80% base call accuracy) (see details in *Methods* in Supplementary File 1).

**Table 1.** Summary of number, base quantity, and length of reads obtained at several steps of the quality filtering pipelines for the tarakihi genome sequences.

Reads	Number of reads	Total number of bases	Minimum read length	Average read length	Maximum read length
Raw Illumina PE reads	425,740,632	63,861,094,800	150	150	150
Quality-filtered Illumina PE reads	405,228,300	60,784,245,000	150	150	150
Uncontaminated Illumina PE reads	367,760,592	55,164,088,800	150	150	150
**Final Illumina PE reads**	366,065,036	54,909,755,400	150	150	150
Raw Nanopore reads cell 1	8,270,853	52,169,467,195	5	6,307.6	1,029,695
Raw Nanopore reads cell 2	7,229,556	47,015,342,634	5	6,503.2	1,035,919
**Final Nanopore reads**	9,178,726	73,394,980,774	450	7,996.2	182,445
HiFi reads	285,997	4,009,988,664	49	14,021.1	27,427
Iso-Seq subreads (4 tissues)	171,924,197	302,196,904,697	51	2,601.15	278,803
**Final Iso-Seq transcripts**	91,602	312,308,038	80	3,409.4	10,426

Reads in bold were the ones used in the final retained (Flye polished) assembly. Final Illumina PE reads have been filtered for quality, DNA contamination, and mitochondrial DNA. Final Nanopore reads have been filtered for quality. Final Iso-Seq CCS transcripts were filtered for quality and repeat transcripts and were nonredundant.

### Genome size, coverage, and heterozygosity estimation postsequencing

**Fig. 2.**
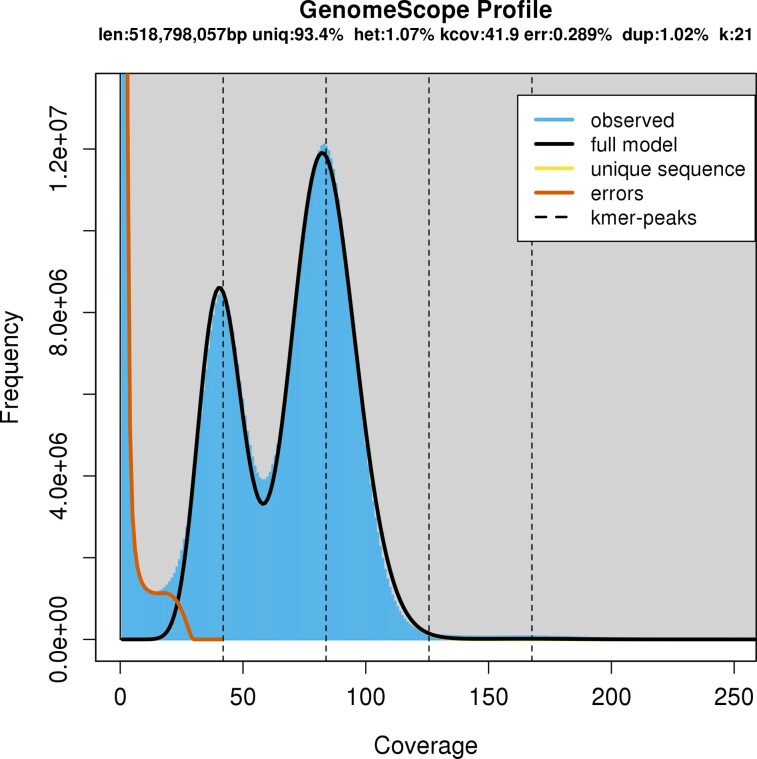
Histogram of 21-mer frequency in illumina reads. Estimation of genome size of tarakihi, heterozygosity, and duplicated regions. The first and second peaks show the *k*-mer frequency of heterozygous and homologous regions, respectively. See Supplementary Fig. 1 in Supplementary File 2 for 17- and 21-mer models.

Genome size and sequencing coverage based on the Illumina sequence reads were performed with a *k*-mer frequency analysis of 17, 21, and 27-mers using jellyfish v2.2.10 (Marçais & Kingsford, 2011) and GenomeScope (Vurture *et al*., 2017) (see details in Supplementary File 1: *Methods*). A haploid genome size of c. 516–520 Mb, with a high heterozygosity level of 1.01–1.07% and a duplication level of 0.98–1.10%, was estimated (Fig. 2, Supplementary Fig. 1 in Supplementary File 2). This estimated genome size was consistent, albeit c. 150 Mb lower than the size estimated presequencing. However, it is common for *k*-mer estimated size and genome assembly size to be smaller than the size estimated with C-value (Austin *et al*., 2017; Jansen *et al*., 2017; Feron *et al*., 2020). The heterozygous coverage of 40× was considered sufficient for performing genome assembly. The heterozygosity of TARdn1 was estimated a second time by calling SNPs from the Illumina reads aligned to the final assembly (see details of parameters in Supplementary File 1: *Methods* and Supplementary Fig. 2 in Supplementary File 2).

### Illumina + nanopore hybrid assembly

*De novo* genome assembly of short- and long-reads was performed with the Maryland Super-Read Celera Assembler pipeline, MaSuRCA (Zimin *et al*., 2013; Zimin, Puiu, *et al*., 2017). This is one of the most common assemblers for performing short- and long-reads hybrid genome assemblies of eukaryotes, with consistently good results across studies (Tan *et al*., 2018; Jiang *et al*., 2019; Thai *et al*., 2019) (see details on the MaSuRCA pipeline in Supplementary File 1: *Methods*). The hybrid Illumina + Nanopore assembly was run on MaSuRCA v3.2.9 (see parameters in Supplementary File 1). MaSuRCA v3.2.9 uses a modified version of the CABOG assembler (Miller *et al*., 2008) for the final assembly of corrected mega-reads. However, later releases of MaSuRCA included the Flye assembler (Kolmogorov *et al*., 2019) as a supposedly faster and more accurate alternative tool for the same step. To compare both methods, a second assembly was run on MaSuRCA v3.4.1 with the same parameters as above, but this time using FLYE_ASSEMBLY = 1. The Flye assembly was subsequently polished with POLCA (Zimin & Salzberg, 2020) as implemented in MaSuRCA v3.4.1 on default settings, using the clean Illumina reads to fix substitutions and indel errors.

### HiFi sequencing and assembly

Assembly of HiFi reads was performed with hifiasm v0.13 (Cheng *et al*., 2021) using default parameters. Another assembly was also tentatively performed with HiCanu as implemented in Canu v2.1.1 (Nurk *et al*., 2020), with an estimated genome size of 600 Mb. However, the read coverage estimated (6.68×) was lower than the minimum coverage allowed by HiCanu (10×), so the assembly could not be completed.

### Quality assessment and comparison of assemblies

After each assembly, basic contiguity statistics were computed with bbmap v38.31 (Bushnell, 2018) script stats.sh. To assess the completeness of the assemblies, the Benchmarking Universal Single-Copy Orthologs (BUSCO) tool v3.0.2 (Simão *et al*., 2015) was used with parameter -sp zebrafish on the Actinopterygii odb9 orthologs set, which contains 4,584 single-copy orthologs that are present in at least 90% of ray-finned fish species.

The quality of the CABOG and Flye assemblies was further compared by mapping clean Illumina reads back to the assemblies themselves with bwa-kit v0.7.15 using bwa mem -a -M. The resulting alignment files were also used to plot Feature Response Curves (FRC) (Vezzi *et al*., 2012b) with FRCbam v5b3f53e-0 (Vezzi *et al*., 2012a). This allowed comparison of the quality of the assemblies without relying on contiguity, by plotting the accumulation of error “features” along the genome (e.g. areas with low or high coverage, numbers of unpaired reads, and misoriented reads). The presence of unmerged haplotigs in the CABOG and the Flye polished assembly was investigated by using minimap v2.16 (Li, 2018) with parameters -ax map-ont –secondary = no to map the clean Nanopore reads back to the assembly and then analyzing the resulting alignment with Purge Haplotigs v1.1.1 (Roach *et al*., 2018) command hist.

The last quality check of the CABOG and Flye polished assemblies was done by plotting assemblies against each other and against two chromosome-level fish assemblies using MashMap 2.0 (Jain *et al*., 2018) with a minimum mapping segment length of 500 bp and a minimum identity of 85% (for comparison between tarakihi assemblies) and 90% (for comparison between different species). To visualize the presence of potential misassemblies on the longest scaffolds, the results from MashMap were used to plot the mappings of these scaffolds between different assemblies with a custom R script (plot_mashmap_scaffolds.R). The first fish chromosome-level assembly used for comparison was the mandarin fish *Siniperca chuatsi* (SinChu7, GCA_011952085.1) because it was the phylogenetically closest chromosome-level assembly (Centrarchiformes, Centrarchoidei) available on NCBI at the time this analysis was performed. The second was the Australasian snapper *Chrysophrys auratus* (SNA1, https://www.genomics-aotearoa.org.nz/data), to compare with a well-curated specimen from a more evolutionarily distant species.

Final visualization of contiguity and completeness of the genome assemblies was generated with assembly-stats v17.02 (Challis, 2017) as implemented in the grpiccoli container (Piccoli, 2021).

### Genome repetitive elements detection

Repetitive elements (REs) in the *N. macropterus* genome were identified both by de novo modeling and based on repeats homology using RepeatModeler v2.0.1 (Flynn *et al*., 2020) and RepeatMasker v4.1.1 (Smit *et al*., 2013) as implemented in Dfam TE Tools container v1.2 (https://github.com/Dfam-consortium/TETools); see Supplementary File 1: *Methods* for more details).

### Iso-Seq analysis

Iso-Seq subreads were processed with the SMRTLink v9.0 Iso-Seq pipeline. Circular consensus sequences were generated and converted into high-quality (predicted accuracy > 0.99), polished, nonredundant isoforms (see details in Supplementary File 1: *Methods*). These isoforms were screened for REs against the *N. macropterus* custom repeat library with RepeatMasker v4.1.1. Transcripts with ≥ 70% bases masked were considered REs. Identified REs were discarded from further analyses using a custom bash script for filtering (Count_filter_N_isoseqrepeats.bash) and categorized using a custom R script (R_charachterize_transcripts.R).

AS events in the repeat-cleaned Iso-Seq reads were counted and classified with SUPPA v2.3 (Trincado *et al*., 2018) with default parameters. These results were compared with reported AS values for other animal species from studies that also used SUPPA on Iso-Seq reads. Results reported were compiled for the zebrafish (*Danio rerio*) (Nudelman *et al*., 2018), the goldfish (*Carassius auratus auratus*) (Gan *et al*., 2021), the Wuchang bream (*Megalobrama amblycephala*) (Chen *et al*., 2021), the whiteleg shrimp (*Litopenaeus vannamei*) (Zhang *et al*., 2019), and the cave nectar bat (*Eonycteris spelaea*) (Wen *et al*., 2018).

### Genome annotation

The *N. macropterus* genome was annotated using the MAKER v2.31.10 (Holt & Yandell, 2011) pipeline, using only the complex repeats for hard masking. SNAP v2013.11.29 (Korf, 2004) and Augustus v3.3.1 (Stanke *et al*., 2004) were used for ab initio gene prediction. Gene predictions were also inferred from the TARdn2 Iso-Seq transcripts and from protein homology by using protein sequences of zebrafish (*D. rerio*), three-spined stickleback (*Gasterosteus aculeatus*), spotted gar (*Lepisosteus oculatus*), Nile tilapia (*Oreochromis niloticus*), medaka (*Oryzias latipes*), Japanese puffer (*Takifugu rubripes*), green spotted puffer (*Tetraodon nigroviridis*), and southern platyfish (*Xiphophorus maculatus*) that were downloaded from Ensembl release version 103 (Kersey *et al*., 2016). Functional annotation of predicted proteins was performed with blast + v2.6.0 against the NCBI nonredundant protein sequences database (NR) and with InterProScan v5.50-84.0 (Jones *et al.*, 2014). Finally, low-quality genes shorter than 50 amino acids were identified with AGAT v0.6.0 (Dainat, 2021) and filtered out. Genes with an incomplete open reading frame (ORF) were flagged. Genome annotation was also inspected visually with JBrowse v1.1.10 (Skinner *et al*., 2009). See all details of the genome annotation pipeline in Supplementary File 1: *Methods*.

## Results

### Genome sequencing

Illumina sequencing reads filtering (i.e. quality, contamination, and mitochondria) resulted in a final data set of 54.91 Gb short reads (Table 1) with a c. 92× depth of coverage. The GC content was 43%, and the overall sequence read quality was high. Both forward and reverse reads passed all the FastQC criteria; that is, they were never flagged for poor quality (Supplementary Fig. 3 in Supplementary File 2). Although there was a small bias in per base sequence contents of the first c. 10 bases, this was expected because of the nonrandom nature of the hexamer priming step during sequencing (Hansen *et al*., 2010). This slight deviation from uniformity in sequence content was not considered an issue because there is no quantitative step involved in the analyses based on the short reads. Nanopore sequencing, filtering, and trimming resulted in 9.18 million reads (73.39 Gb), or 122× coverage, with an average read length of 8 Kb (Table 1), a mean read quality of 7.9, and an N50 length of 9.5 Kb. A total of 285,997 CCS HiFi reads (4.01 Gb) and 91,602 repeat-free, nonredundant, high-quality Iso-Seq transcripts (312.31 Mb) were also obtained.

### Assemblies comparison and quality assessment

The Flye assembly reduced the number of scaffolds by more than half compared with the CABOG assembly (Table 2). The scaffold N50 length of the Flye assembly was almost twice as long, and the number of complete BUSCOs was higher. The Flye assembly size was also more consistent with the haploid genome size pre-estimated by *k*-mer counting (c. 520 Mb) than the CABOG assembly size. Interestingly, the Flye assembly also corrected a misassembly of the first scaffold of the CABOG assembly (see below). Polishing the Flye assembly resulted in the correction of 43,080 substitution errors and 42,783 deletion errors. The polished assembly had the same number of scaffolds and contigs, but a few hundred fewer bases, and one missing BUSCO was recovered into an additional single-copy BUSCO. The hifiasm assembly performed on the HiFi reads did not produce satisfactory results compared with the Illumina + Nanopore hybrid assemblies, with 6–10 times more scaffolds, an N50 length 50 times smaller, and a BUSCO completeness lower than 90%. This was most probably due to the low coverage of HiFi reads (c. 6.5×) used for this sequencing trial.

**Table 2.** General statistics of the four assemblies produced for the tarakihi genome sequence.

Reads type	PacBio HiFi reads	Illumina + Nanopore reads		
	hifiasm	MaSuRCA-CABOG	MaSuRCA-Flye	MaSuRCA-Flye polished
Genome assembly				
ȃScaffold assembly size (bp)	778,095,731	608,975,097	567,903,348	567,902,715
ȃTotal number of scaffolds	13,511	2,696	1,214	1,214
ȃLongest scaffold (bp)	469,394	18,930,378	13,913,512	13,913,694
ȃScaffold N50/L50	67.836 Kb/3,650	1.87 Mb/69	3.37 Mb/45	3.37 Mb/45
ȃScaffold N90/L90	30.868 Kb/10,456	140.52 Kb/535	437.51 Kb/219	437.54 Kb/219
ȃProportion of gap sequences (%)	0.001	0.002	0.001	0.001
ȃContigs size (Mb)	778.096	609.964	567.900	567.900
ȃTotal number of contigs	13,511	2,809	1,245	1,245
ȃContig N50/L50	67.836 Kb/3,650	1.79 Mb/74	2.94 Mb/52	2.94 Mb/52
ȃContig N90/L90	30.868 Kb/10,456	137.36 Kb/556	429.99 Kb/242	429.98 Kb/242
ȃA/T/G/C/bases (%)	28.17/28.14/21.84/21.85	28.06/28.13/21.91/21.90	28.10/28.15/21.87/21.88	28.10/28.15/21.87/21.88
ȃGC standard deviation (%)	2.13	5.87	3.87	3.87
Genome completeness (4,584 Actinopterygii BUSCOs)				
ȃComplete BUSCOs (%)	88.8	97.6	97.7	97.8
ȃComplete single-copy BUSCOs (%)	57.3	92.9	95.1	95.2
ȃComplete duplicated BUSCOs (%)	31.5	4.7	2.6	2.6
ȃFragmented BUSCOs (%)	3.5	0.8	0.8	0.8
ȃMissing BUSCOs (%)	7.7	1.6	1.5	1.4

The MaSuRCA-Flye polished assembly (in bold) yielded the best results and was retained for all subsequent analyses.

**Fig. 3.**
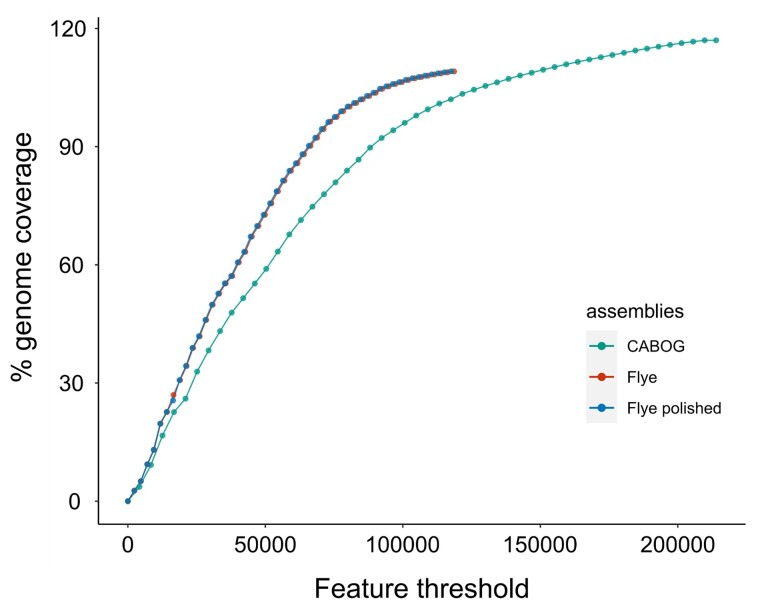
FRC curves for the CABOG, Flye, and Flye polished assemblies of tarakihi. The *Y*-axis represents the cumulative size of the assembly and the *X*-axis is the cumulative number of potential errors (i.e. “features”). Assemblies for which the curves are steeper are considered more accurate.

**Fig. 4.**
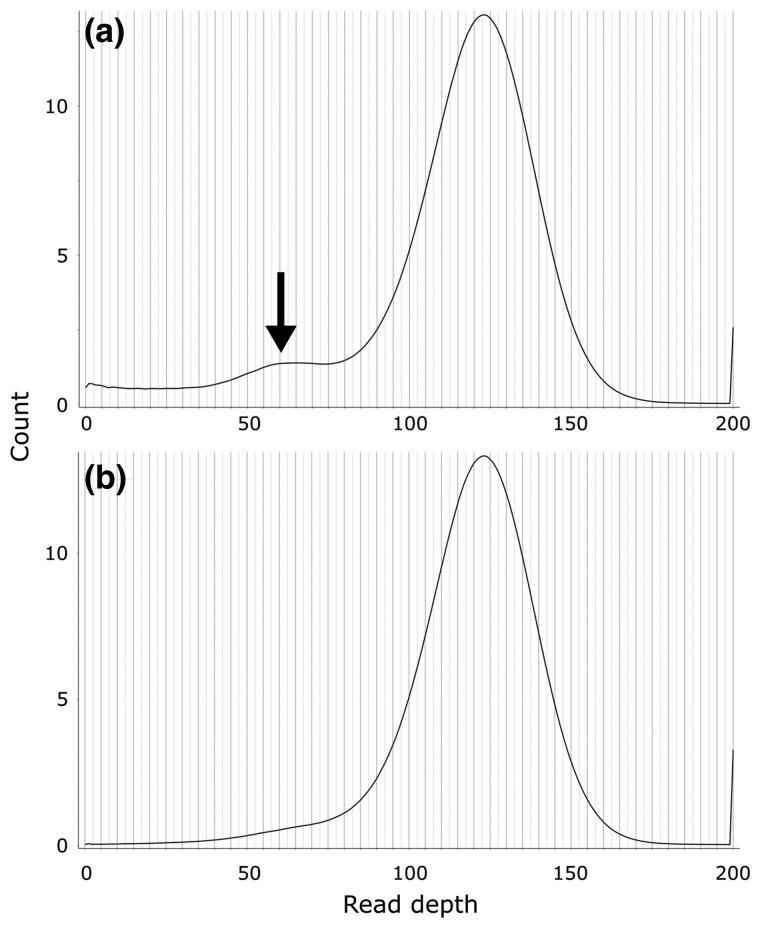
Read depth histograms of the tarakihi genome assemblies contigs, obtained by mapping the clean Nanopore reads back to the assembly. A unimodal distribution with a peak equal to the sequencing reads depth is expected for a haplotig-free assembly. Another peak at half the sequencing reads depth (arrow) is indicative of the presence of unmerged haplotigs. a) CABOG assembly and b) Flye polished assembly.

Approximately 99.7% of Illumina reads could be mapped back to the CABOG assembly, and 99.8% to both Flye assemblies, making the Flye assemblies slightly more accurate according to that metric. The Flye polished assembly had a slightly higher proportion of “proper-pairs” reads mapped (86.23%) than the unpolished assembly (85.7%). FRC curves showed that both Flye assemblies were more accurate than the CABOG assembly (Fig. 3). Moreover, while both the unpolished and polished Flye assemblies produced a very similar curve, for the same genome coverage, the polished Flye assembly always had a slightly lower number of cumulative errors than the unpolished assembly (Supplementary Fig. 4 in Supplementary File 2). While there was evidence of the presence of unmerged haplotigs in the CABOG assembly (Fig. 4a), no evidence was detected in the Flye polished assembly (Fig. 4b); thus, a filtering step was not required. Trailing Ns were not present in the Flye polished assembly either.

**Fig. 5.**
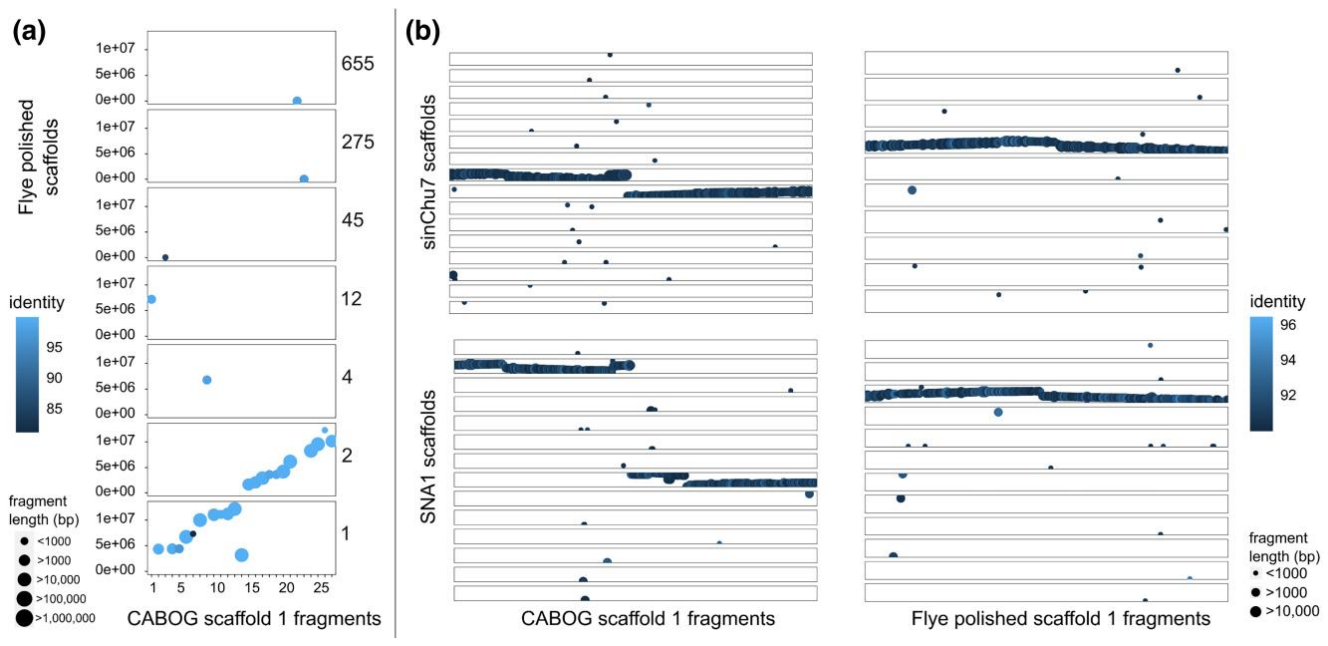
Scaffolds plotted against total assemblies based on identity results from MashMap with minimum mapping region (i.e. “fragments”) length of 500 bp. Each horizontal box is a scaffold of the reference on which the query scaffolds are mapped according to a given identity threshold. Mapped regions are ordered by base coordinate along the query scaffold on the *X*-axis, and the reference scaffolds on the *Y*-axis. a) CABOG assembly scaffold 1 mapped to the total polished Flye assembly, with corresponding Flye scaffold numbers reported on the right and b) CABOG and Flye assemblies scaffold 1 mapped to the *Siniperca chuatsi* and *Chrysophrys auratus* chromosome-level assemblies.

Interestingly, the longest scaffold of the CABOG assembly, scaffold 1, was 5 Mb longer than the longest scaffold of the Flye assembly (Table 2). Between-scaffolds alignment scores obtained from MashMap (Supplementary Fig. 5 in Supplementary File 2) were used to visualize a potential misassembly at that scaffold. The longest scaffold of the CABOG assembly corresponded indeed to the two longest scaffolds of the polished Flye assembly, scaffolds 1 and 2 (Fig. 5a). The CABOG scaffold 1 is highly likely to have been misassembled since it also corresponds to two long regions in two different linkage groups (i.e. chromosomes) in chromosome-level assemblies of both *S. chuatsi* and *C. auratus*. This is not the case for scaffold 1 in the polished Flye assembly (Fig. 5b). This supported the interpretation that the “correct” longest scaffold is the one from the polished Flye assembly.

**Fig. 6.**
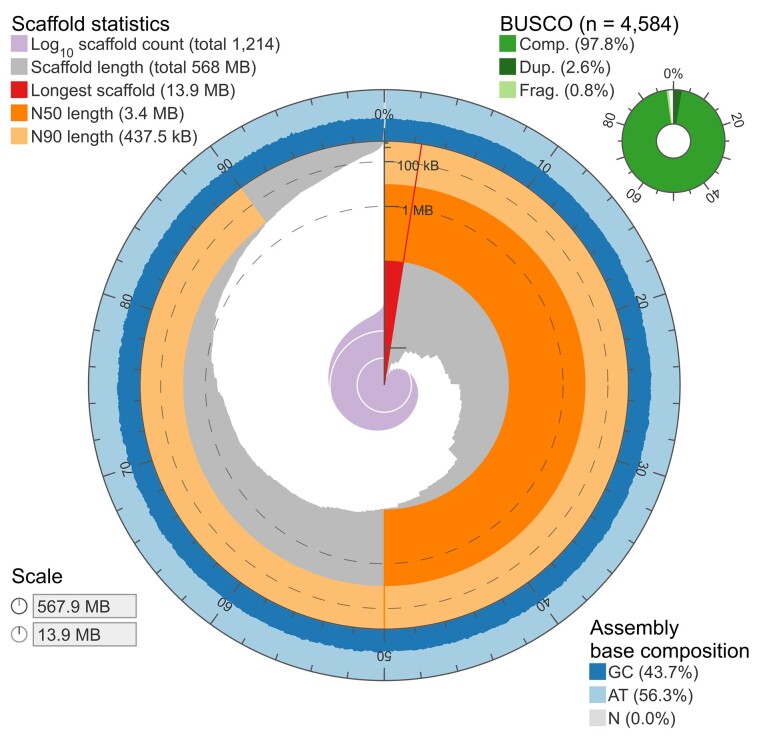
Visualization of contiguity and completeness of the final tarakihi assembly. The contiguity is visualized in a circle representing the full assembly length of c. 568 Mb. The longest scaffold was 13.9 Mb. There were very few scaffolds (c. 2%) shorter than 100 Kb in length and the GC content was uniform throughout. See Supplementary Fig. 6 in Supplementary File 2 for a comparison with the three other assemblies that were not retained.

The Flye polished assembly provided the best results and thus was used in all subsequent analyses. This final genome assembly consisted of 567,902,715 bases in 1,214 scaffolds, with a scaffold N50 length of 3.37 Mb and a proportion of gaps of 0.001% (Table 2, Fig. 6). The base composition was A: 28.10%, T: 28.15%, G: 21.87%, C: 21.88%, and overall standard deviation of GC content was 3.87%. The BUSCO completeness was very good overall, with >95% of the single-copy Actinopterygii orthologs retrieved in the final assembly (Table 2; Fig. 6). The contiguity and completeness were high compared with those of other Illumina + Nanopore hybrid assemblies (Table 3).

**Table 3.** Comparison of the contiguity and completeness of genomes that were assembled using a hybrid approach including only short Illumina reads and long Nanopore reads.

Species	Genome (total scaffolds) length (Mb)	Number of scaffolds	Scaffold N50 length (Mb)	Complete BUSCOs	Protein-coding gene models	Functionally annotated genes
Tarakihi	568	1,214	3.4	97.80%	20,327	19,823
Murray cod	633	18,198	0.1	94.20%	26,539	25,607
Clownfish	881	6,404	0.4	96.30%	27,420	26,211
Danionella translucida	735	27,639	0.3	91.50%	24,097	21,491
Snout otter clam	544	622	2.1	95.80%	26,380	23,701
Indian blue peacock	915	15,025	0.2	Not reported	23,153	21,854

All fish genome assemblies that corresponded to the criteria are reported [Murray cod (*Maccullochella peelii*): Austin *et al*., 2017; clownfish (*Amphiprion ocellaris*): Tan *et al*., 2018; *Danionella translucida*: Kadobianskyi *et al*., 2019] and two selected additional species have been included for comparison with other groups of organisms [Mollusk, snout otter clam (*Lutraria rhynchaena*): Thai *et al*., 2019; bird, Indian blue peacock (*Pavo cristatus*): Dhar *et al*., 2019].

### Estimation of heterozygosity

Variant calling of Illumina reads against the polished assembly resulted in a total of 3,654,819 SNPs. By dividing this number by the size of the genome, this corresponded roughly to a heterozygosity level of 0.64%. This is lower than the level estimated by *k*-mer frequency (c. 1.00%). However, it is common for heterozygosity estimated by k-mer frequency to be lower than estimated by called SNPs, because the SNP calling approach is more conservative (Thai *et al*., 2019). Nevertheless, the heterozygosity estimated for TARdn1 is one of the highest reported for fish species. To our knowledge, this is the highest heterozygosity estimated for a fish through *k*-mer analysis, with other reported values ranging from 0.1% (Tibetan loach *Triplophysa tibetana* and Murray cod *Maccullochella peelii*) to 0.9% (Java medaka *Oryzias javanicus*) (Vij *et al*., 2016; Austin *et al*., 2017; Gong *et al*., 2018; Ge *et al*., 2019; Nguinkal *et al*., 2019; Yang *et al*., 2019; Lu *et al*., 2020; Takehana *et al*., 2020; Zhang *et al*., 2020; Zheng *et al*., 2021). Even the heterozygosity estimated through SNPs (0.64%) is high compared with estimations from other fishes using the same method [e.g. large yellow croaker: 0.36% (Wu *et al*., 2014), grass carp: 0.25% (Wang *et al*., 2015)]. This result is even more striking in that the variant analysis was very stringent in our case by retaining only high-quality bi-allelic SNPs. This reinforces the recent findings that *N. macropterus* is a species with a historically large population that displays a particularly high genetic diversity (Papa, Halliwell, *et al*., 2021).

### Repetitive elements and genes annotation

REs represented 30.45% of the genome or a total of 172,911,032 bp. Although the proportion of REs in fish genomes can vary greatly at scales from 10 to 60% (Yuan *et al*., 2018), the proportion of repeat elements in *N. macropterus* is at par with the proportion observed in other Centrarchiformes [Largemouth bass (*Micropterus salmoides*): 33.79%; Big-eyed mandarin fish (*Siniperca knerii*): 26.55% (Lu *et al*., 2020; Sun *et al*., 2021)] and for Perciformes in general (Yuan *et al*., 2018). Of the REs known in the databases, interspersed repeats accounted for 27.62% of the genome, including 10.87% of DNA transposons and 6.17% of retro-elements (long interspersed nuclear elements (LINEs), long terminal repeat retrotransposons (LTR), short interspersed nuclear elements (SINEs), and Penelope (PLE), in that order. The rest of the repeat elements consisted of simple sequence repeats (Supplementary Table 2 in Supplementary File 2). After filtering for length, the final predicted gene set included 20,169 protein-coding genes with a mean length of 13,832 bp, among which 95.5% had a maximum Annotation Edit Distance (AED) < 0.5. An AED value of 0 indicates an exact match between the intron/exon coordinates of an annotation and the aligned transcriptome and proteins evidence, while an AED of 1 indicates no support from evidence. The mean exon length was 229 bp, and the mean intron length in CDS was 1,184 bp. More than 98% of the genes were functionally annotated by at least one of the two methods used (blastp, 98.2%; InterProScan, 82.8%).

### Iso-Seq analysis

**Fig. 7.**
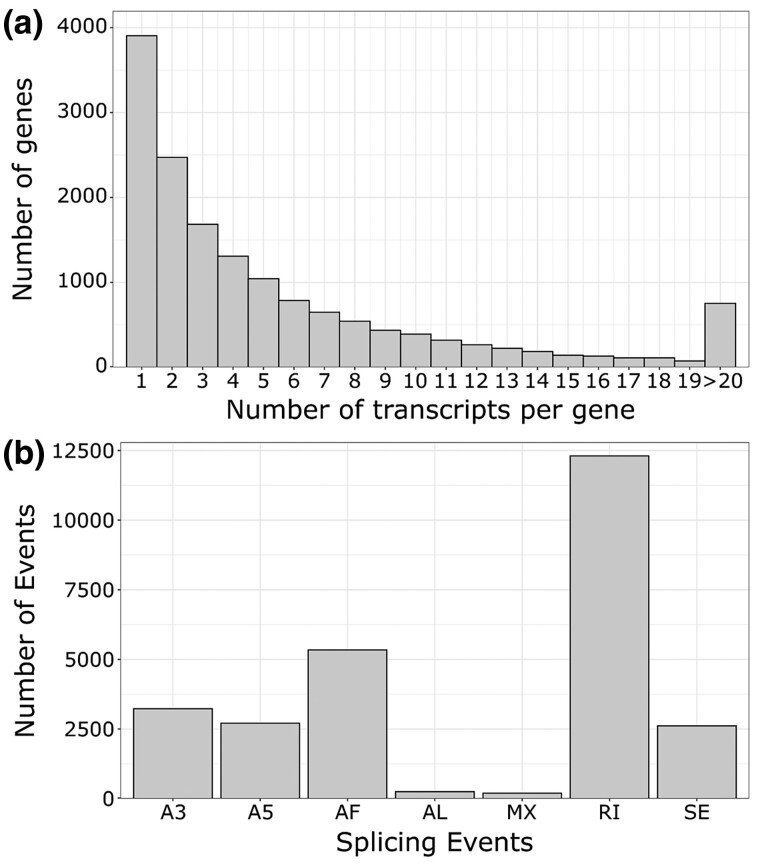
Alternative transcripts metrics in the tarakihi transcriptome: a) number of unique alternative transcripts per gene and b) classification and frequency of alternative splicing events. A5/A3: Alternative 5′/3′ splice sites. AF/AL, alternative first/last exons; MX, mutually exclusive exons; RI, retained intron; SE, skipping exon.

Of the 93,949 full-length polished, nonredundant Iso-Seq transcripts, 2,347 were classified as REs and were filtered out from downstream analyses. For each of these RE transcripts, the main RE elements included DNA elements (801), LINEs (639), LTRs (464), SINEs (94), rRNAs (47), low complexity/simple repeats (33), rolling circles (26), satellites (16), and retroposons (2), as well as one LINE/LTR hybrid, and 224 unknown RE. The final non-RE Iso-Seq data set included 91,313 unique transcripts from 15,515 genes. The mean transcript per gene ratio was 5.89, with a median of 3 and a maximum of 211 (Fig. 7a). This is higher than the values recently reported for humans (3.62) and two species of bats (1.92 and 1.49), but lower than for pharaoh ants (9) (Wen *et al*., 2018; Gao *et al*., 2020). Less than 5% of genes had more than 20 different transcripts. The predicted proteins of both genes that produced the most transcripts (respectively 211 and 164 transcripts) were collagen alpha chains isoforms (XP_006787735.1: collagen alpha-2(I) chain-like isoform X2, XP_020490299.1: collagen alpha-1(V) chain-like isoform X1), implicated in the structural integrity of the cellular matrix (GO:0005201).

**Fig. 8.**
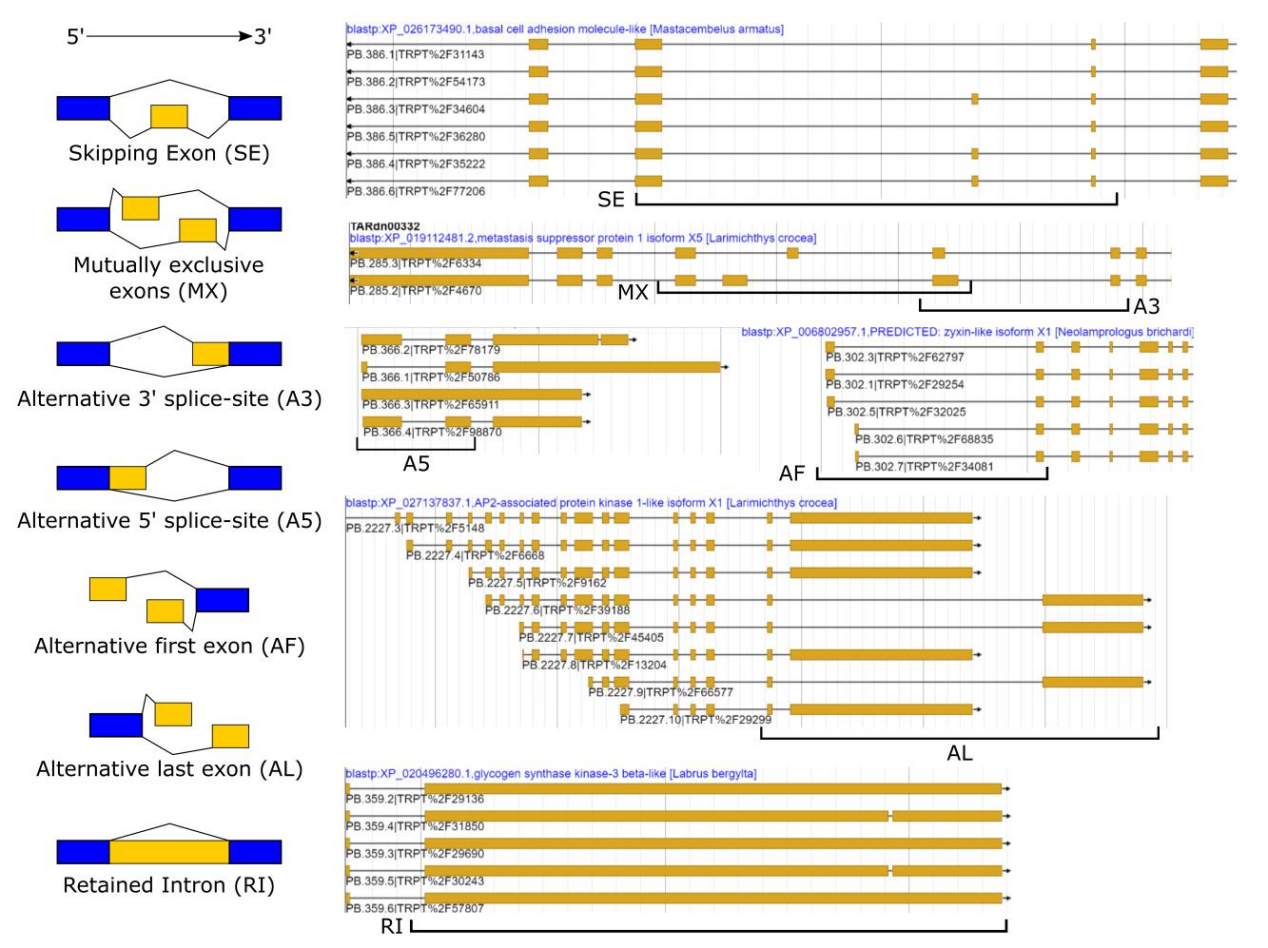
The seven types of alternative splicing events classified in the tarakihi transcriptome, with examples of each event class as visually shown in the annotation of the genome.

**Fig. 9.**
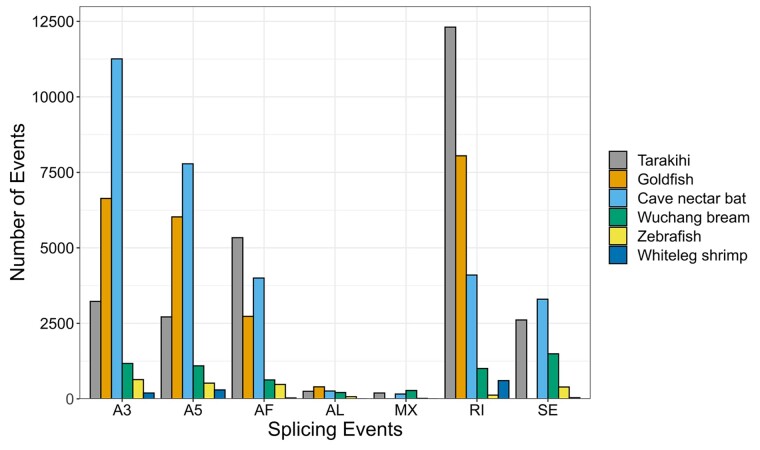
Comparison of alternative splicing event counts between tarakihi and five other animal species from other Iso-Seq AS studies. MX and SE events were not reported in the goldfish study.

A total of 26,644 AS events were detected in the tarakihi transcriptome (Fig. 7b). The most frequent AS event was the retention of intron (46%), while “alternative last exons” and “mutually exclusive exons” were the rarest (<1% each). Some examples of these AS events were visualized in the tarakihi genome (Fig. 8). Comparison of the frequency of AS events in the tarakihi with other species showed that the trends are globally similar across organisms (Fig. 9). Most organisms show relatively high occurrences of retained introns (RIs), alternative 3' and 5'; splice sites (A3 and A5), alternative first exons (AF), and to a lesser degree skipping exons (SE), compared with alternative last exons (AL) and mutually exclusive exons (MX). Figure 9 also shows that tarakihi, goldfish, and cave nectar bat may have a better representation of the AS events proportions owing to a much deeper coverage than the Wuchang bream, zebrafish, and whiteleg shrimp (although values for MX and SE were not reported for the goldfish study). While it is the most common AS event in both tarakihi and goldfish, the proportion of RI events is much higher in tarakihi than in the proportion of other events. While intron retention was thought until recently to be the least prevalent AS form in animals, it is now clear that this is not the case (as shown in the studies in Fig. 9 but also e.g. Wang *et al*. 2019; Gao *et al*. 2020). RI events are widely used across organisms to tune down the levels of transcription of some genes in cells and tissues depending on their function (Braunschweig *et al*., 2014).

### Genome size

The size of the tarakihi genome was consistent with values for fish genomes that have been reported so far. A recent review of publicly available fish genome assemblies (comprising 244 species) showed that the average genome length of fish is 872.64 Mb but varies between c. 300 Mb and c. 4.5 Gb (Fan *et al*., 2020). The genome size of *N. macropterus* (568 Mb) is several hundred Mb shorter than the two other published Centrarchiforme genomes, for the largemouth bass *M. salmoides* (964 Mb) and the big-eye mandarin fish *S. knerii* (732.1 Mb) (Lu *et al*., 2020; Sun *et al*., 2021). However, *N*. *macropterus* is still evolutionarily far apart from these two species. The largemouth bass and the big-eye mandarin fish both belong to the Centrarchoidei suborder, which is thought to have split from Cirrhitioidei at least 70 million years ago (Sanciangco *et al*., 2016).

## Discussion

The advances in DNA sequencing technologies have made it clear how valuable reference genome assemblies are for the study of biology and conservation, resulting in a global effort to assemble the genomes of as many organisms as possible (Koepfli *et al*., 2015; Worley *et al*., 2017; Fan *et al*., 2020). Here, we present the first genome assembly of the tarakihi, a valuable commercial fisheries species, and the first representative out of the c. 60 species of the Cirrhitioidei suborder to have a whole genome sequenced. While performing a hybrid assembly of Illumina and Nanopore reads with the latest tools led to a highly contiguous assembly with high gene completeness, this could be still improved in the future by adding Hi-C data to scaffold it to a chromosome-level assembly (Whibley *et al*., 2021). Moreover, while PacBio HiFi data were a very new and still relatively expensive technology at the time of data collection, this will probably replace the short and long-reads hybrid assembly method as the optimal genome assembly strategy by offering the best of both worlds (long reads and high quality) and allowing phasing of genomes. Nonetheless, the present genome assembly has already been successfully used as a resource for population structure analyses and the study of adaptive selection (Papa, Morrison, *et al*., 2022) and in the context of comparative genomics (Papa, Wellenreuther, *et al*., 2022). The highly accurate transcriptome will also surely be a valuable resource for future studies.

## Supplementary Material

jkac315_Supplementary_Data

## Data Availability

All genomic sequences and associated metadata are deposited on the Genomics Aotearoa repository (https://repo.data.nesi.org.nz/) under projects “Tarakihi genome” (https://doi.org/10.57748/aabn-5y92) and “Tarakihi transcriptome” (https://doi.org/10.57748/xkby-3t51). All scripts used in the analyses are openly available on GitHub at https://github.com/yvanpapa/tarakihi_genome_assembly. Supplemental material available at G3 online.

## Notes

*Communicating editor: A. McCallion*
