# Supplementary material for: Genome assembly and isoform analysis of a highly heterozygous New Zealand fisheries species, the tarakihi (Nemadactylus macropterus)
Source: G3 (Bethesda). 2022 Dec 8;13(2):jkac315. doi: 10.1093/g3journal/jkac315 (PMC9911067; doi:10.1093/g3journal/jkac315)
Supplement: jkac315_Supplementary_Data [file jkac315_supplementary_data.zip › File_S1_G3-2022-403723.docx]

# **Supplementary Material**

# Genome assembly and isoform analysis of a highly heterozygous New Zealand fisheries species, the tarakihi (*Nemadactylus macropterus*)

Yvan Papa, Maren Wellenreuther, Mark A. Morrison, Peter A. Ritchie

## Materials and Methods

### Specimens collection

Both tarakihi specimens were identified as male by observation of the gonads. The TARdn2 specimen was collected for tissue sampling after being filleted by the fisherman.

### Genome size estimation pre-sequencing

To estimate the size of the *N. macropterus* genome and ensure there was a sufficient amount of DNA sequencing for adequate coverage, genome information from closely related species was assessed. As of October 2018, only two other Centrarchiformes genome assemblies were deposited in NCBI at the scaffold level (accession numbers: GCA_002120245.1 (Murray cod, *Maccullochella peelii*), and GCA_003416845.1 (barred knifejaw, *Oplegnathus fasciatus*)), which had genome lengths of 633.24 and 766.3 Mb. Moreover, the species closest to *N. macropterus* for which genome size was estimated on the Animal Genome Size Database (http://www.genomesize.com) was the red morwong *Cheilodactylus fuscus*, with a C-value of 0.72, or approximately 700 Mb. The genome size of *N. macropterus* was thus estimated to be about 700 Mb. The quantity of Illumina and Nanopore bases to be sequenced was tuned for a deep 85× Illumina coverage (c. 60 Gb) and 140× Nanopore coverage (c. 100 Gb), following sequencing provider recommendations.

### Library preparations and sequencing

Library preparations, sequencing, and the first filtering step (except for Nanopore reads) were performed by the sequencing providers. For Illumina reads, DNA samples were sheared with Bioruptor® Pico system (Diagenode) for a fragment insert size of 350+/-50 bp, and a PCR-free library was obtained with NEBNext® Ultra™ II DNA Library Prep Kit for Illumina (New England Biolabs). Approximately 200 million of 150 bases pair-end reads were generated using the HiSeq X System (Illumina). Raw Illumina reads were filtered by discarding read pairs if (1) one read contained some adapter contamination for more than five nucleotides; (2) more than 10% of bases were uncertain in either read of the pair; or (3) the proportion of bases with Quality Value ≤ 19 was over 50% in either read. For Nanopore library preparation, large size DNA fragments were selected by automated gel electrophoresis with BluePippin (Sage Science) followed by enrichment and purification using beads. Fragmented DNA was then end-repaired, A-tailed, and purified, and adapter ligation was done using the Ligation Sequencing Kit 1D 108 (Oxford Nanopore Technologies). The resulting DNA library of 20–40 Kb fragments was then loaded into two flow cells for real-time single-molecule sequencing on PromethION (Oxford Nanopore Technologies). Reads were base-called from their raw FAST5 files using Albacore 2.0.1 (https://community.nanoporetech.com). The HiFi library was prepped with SMRTbell® Express Template Prep Kit 2.0 (Pacific Biosciences) and CCS was performed on one-third of an SMRT Cell 8M with a PacBio Sequel II sequencer. ZMWs were filtered to retain a minimum of three passes and a predicted quality value (RQ) of 99. Four Iso-Seq libraries of 0–5 kb insert sizes (one per tissue) were generated using the SMRTbell® Express Template Prep Kit 2.0. The multiplexed libraries were sequenced on one SMRT Cell 8M with a PacBio Sequel II sequencer, resulting in 3.6 million polymerase reads from which sub-reads were extracted.

### Mitogenome assembly and exclusion

Illumina reads filtered for quality and contamination were mapped against the Peruvian morwong (*Cheilodactylus variegatus*) complete mitochondrial sequence retrieved from GenBank (accession number: KP704218.1) with Geneious v11.04 (Kearse et al., 2012) using five iterations of the default mapper set to the highest sensitivity. The extracted consensus sequence resulted in a 16,650 bp assembly of the *N. macropterus* mitogenome. The mitogenome was then annotated using the MitoAnnotator web interface (Iwasaki et al., 2013). Sequences of mitochondrial origin were then filtered out of the Illumina reads as follows: first, bwa-kit v0.7.15 (Li & Durbin, 2009) was used to align the Illumina reads to the indexed *N. macropterus* reference mitogenome with default parameters. Among other aligners, the BWA-MEM algorithm (Li, 2013) was selected because it is the most accurate for this type of short-read data (Keel & Snelling, 2018). In the resulting SAM alignment, 0.46% of reads mapped to the mitogenome. Then, all the reads from the alignment that did not map to the mitogenome were extracted to a new mitochondria-free alignment using SAMtools v1.9 (Li et al., 2009) view with parameters -b -f 4, sorted by name, and finally converted back to FASTQ paired-end reads with bedtools v2.27.1 (Quinlan & Hall, 2010).

### Genome size, coverage, and heterozygosity estimation post-sequencing

Genome size and sequencing coverage based on the Illumina sequence reads was performed with a *k*-mer frequency analysis. Total number of 17, 21, and 27-mers were counted with jellyfish v2.2.10 (Marçais & Kingsford, 2011) command count and the resulting histograms were computed with command histo. The histograms were analyzed with GenomeScope (Vurture et al., 2017).

### Nanopore reads sequencing and filtering

Quality control of the raw Nanopore reads was performed with NanoPack v1.0.0 (De Coster et al., 2018) using NanoStat on both FASTQ reads and Albacore summary files. Nanopore reads were filtered and trimmed with NanoFilt by applying a minimum length cut-off of 500 bases (Tan et al., 2018), a minimum average read quality score of 7 (c. 80% base call accuracy), and removing the first 50 nucleotides following the author’s recommendations (De Coster, 2017). Given that quality values based on summary files were slightly lower overall than when based on reads (as expected, cf. github.com/wdecoster/nanofilt), the quality filtering was done based on the summary file values being more stringent. Filter-trimmed reads from both cells were merged into a single FASTQ file.

### Illumina + Nanopore hybrid assembly

*De novo* genome assembly of short and long reads was performed with the Maryland Super-Read Celera Assembler pipeline, MaSuRCA (Zimin et al., 2013, 2017). In brief, MaSuRCA typically works as follows: Illumina paired-end short reads are first assembled into non-ambiguous super-reads, which are then mapped to Nanopore reads to further assemble them in long, high-quality pre-mega-reads. If there are gaps between mega-reads in respect to their mapping to the Nanopore reads, these gaps are filled with the Nanopore read sequence only if the Nanopore read stretch meets some minimum criteria of coverage and quality to produce the mega-reads. If there are still gaps that cannot be merged between mega-reads owing to poor quality of the Nanopore sequence, regions flanking these gaps are linked together as linking pair mates. The mega-reads and linking pairs are then assembled with either CABOG or Flye (see below).

Before assembly, the filtered Illumina reads were not trimmed or edited as per MaSuRCA author recommendation (https://github.com/alekseyzimin/masurca). The hybrid Illumina + Nanopore assembly was run on MaSuRCA v3.2.9 with recommended parameters, automatic *k*-mer size computation, and a jellyfish hash size of 20,000,000,000 (PE = pe 350 50, NANOPORE, EXTEND_JUMP_READS = 0, GRAPH_KMER_SIZE = auto, USE_LINKING_MATES = 0, USE_GRID = 0, GRID_BATCH_SIZE = 300000000, LHE_COVERAGE=25, MEGA_READS_ONE_PASS=0, LIMIT_JUMP_COVERAGE = 300, CA_PARAMETERS = cgwErrorRate = 0.15, KMER_COUNT_THRESHOLD = 1, CLOSE_GAPS = 1, NUM_THREADS = 32, JF_SIZE = 20000000000, SOAP_ASSEMBLY = 0).

### HiFi sequencing and assembly

HiFi reads were converted from BAM to FASTA and FASTQ with SMRTLink v9.0 (PacBio, 2020) bam2fastx. The primary contigs of hifiasm assembly were extracted from the GFA graph and converted to FASTA with command awk '/^S/{print ">"$2;print $3}'.

### Quality assessment and comparison of assemblies

Length, GC content, and GC skew of scaffolds in all assemblies were also reported with seqkit v0.10.1 (Shen et al., 2016) command fx2tab. Augustus v3.3.1 (Stanke et al., 2004), NCBI blast+ v2.7.1 (Camacho et al., 2009), hmmer v3.2.1 (Eddy, 2011), and R v3.6.0 (R Core Team, 2020) were also required to run the BUSCO shell script. The presence of trailing Ns in the Flye polished assembly was tested by using seqkit v0.10.1 command -is replace -p "^n+|n+$" -r "" and comparing the input and the output.

### Estimation of heterozygosity post-assembly

The heterozygosity of TARdn1 was estimated a second time by calling SNPs from the Illumina reads aligned to the final assembly. The reads were mapped to the polished assembly with bwa-kit v0.7.15 using the command bwa mem -a -M. Duplicates were marked with picard v2.18.20 (Broad Institute, 2019) MarkDuplicates. SNPs were called using bcftools v1.9 (Li, 2011) commands mpileup (–C50 –q10 –incl-flags 2) and call (-m -- variants-only -- skip-variants indels). To filter for good quality SNPs, variants’ depth distribution was plotted. The modal depth of coverage was 82, with an increase in steepness starting at c. 20 and a decrease starting at c. 120 (Figure S2 in File S2). Consequently, the final SNP set was filtered with vcftools v0.1.16 (Danecek et al., 2011) for a minimum reference allele frequency of 0.25, a genotype depth of minimum 20 and maximum 120, and a minimum site quality of 20.

### Genome repetitive elements detection

Repetitive elements (RE) in the *N. macropterus* genome were identified both by *de novo* modeling and based on repeats homology. RepeatModeler v2.0.1 (Flynn et al., 2020), as implemented in Dfam TE Tools container v1.2 (https://github.com/Dfam-consortium/TETools), was used to identify repeat models *de novo* using parameter -LTRStruct to include the detection of long terminal repeat retrotransposons. For the homology-based library, RepeatMasker v4.1.1 (Smit et al., 2013) tool famdb.py was used to obtain known Actinopterygii repeats from the combined total Dfam v3.3 (Storer et al., 2021) and RepBase RepeatMasker Edition v20181026 (Bao et al., 2015) databases, using parameters --ancestors –descendants --include-class-in-name --add-reverse-complement. Both *de novo* and homology-based repeat libraries were then concatenated in a custom repeat library for *N. macropterus*. The genome assembly sequences were then mapped against the custom repeat library with RepeatMasker v4.1.1 (-gff -xsmall) to classify repeat regions, create a repeat annotation file, and produce a “soft-masked” (i.e. masked bases in lower case) genome assembly. An alternate “hard-masked” assembly was also created by converting lower cases in the soft-masked assembly into Ns.

### Iso-Seq analysis

Iso-Seq sub-reads were processed with the SMRTLink v9.0 Iso-Seq pipeline. Circular consensus sequences were generated from the sub-reads with command ccs using a minimum read quality (RQ) of 0.9. Clontech and NEB primers removal and de-multiplexing were performed using lima with parameters --isoseq --dump-clips --peek-guess. Poly-A tails were trimmed and concatemers were removed with isoseq3 refine. At that point, BAM files containing sequence reads from the four tissues were merged into one. Clustering and polishing of full-length reads were performed with isoseq3 cluster and parameter --use-qvs to obtain a dataset of high-quality isoforms with a predicted accuracy > 0.99. These high-quality polished isoforms were then aligned to the unmasked *N. macropterus* genome with pbmm2 (--preset ISOSEQ --sort). Subsequently, redundant isoforms were collapsed into non-redundant transcripts loci using the command collapse.

### Genome annotation

The unmasked *N. macropterus* genome was annotated using the MAKER v2.31.10 (Holt & Yandell, 2011) pipeline. First, the simple repeats were filtered out of the repeats annotation file with a custom bash script (rm_simple_repeats.bash) to retain only complex repeats. Only complex repeats were kept because MAKER will hard-mask every region provided in the repeats annotation file before running, discarding them from the gene detection process. However, simple repeats should be available for gene annotation because low-complexity regions are expected within many genes. Hard-masking only complex repeats regions as a first step allows MAKER to subsequently identify and soft-mask the simple repeats regions internally. Gene matches that start in a non-masked region but extend into a soft-masked region can then be taken into account in the gene detection process. A first round of MAKER was run on the unmasked genome using the high-quality, non-redundant, non-repetitive Iso-Seq transcripts to infer gene predictions (est2genome = 1). For repeat masking during this step, the complex repeats GFF file was provided for hard masking and only simple repeats were annotated (model_org = simple). All GFF and FASTA outputs were then merged with ggf3_merge and fasta_merge. Training files for the *ab initio* gene predictors SNAP v2013.11.29 (Korf, 2004) and Augustus v3.3.1 (Stanke et al., 2004) were generated based on round 1 results. For SNAP, only gene models with a maximum Annotation Edit Distance (AED) of 0.25 and a minimum protein length of 50 were used. For Augustus, all the regions that contain mRNA annotations, including the 1,000 surrounding bp, were extracted to a FASTA file using a custom bash script (augustus_rndx.bash). BUSCO v3.0.2 was then run in “genome” mode on the FASTA file using the Actinopterygii odb9 orthologs set, the zebrafish as the initial HMM model, and parameter --long to self-train Augustus. MAKER was then run a second time using SNAP and Augustus training files, as well as the Iso-Seq transcriptome and repeats alignments as evidence (est2genome = 0). For this, all lines containing “est2genome” and “repeat” in the merged GFF from round 1 were extracted and copied in two files that were provided as evidence with the parameters est_gff and rm_gff, respectively. Additionally, gene predictions were also inferred from protein homology during this round (protein2genome = 1), by using protein sequences of zebrafish (*Danio rerio*), three-spined stickleback (*Gasterosteus aculeatus*), spotted gar (*Lepisosteus oculatus*), Nile tilapia (*Oreochromis niloticus*), medaka (*Oryzias latipes*), Japanese puffer (*Takifugu rubripes*), green spotted puffer (*Tetraodon nigroviridis*), and southern platyfish (*Xiphophorus maculatus*) that were downloaded from Ensembl release version 103 (Kersey et al., 2016). After that, SNAP was trained again using the results from round 2, and a third run was performed by using the *ab initio* training files, as well as the extracted repeats, Iso-Seq, and protein homology GFF files as evidence. Genes were renamed with MAKER maker_map_ids and map_x_ids.

All proteins predicted from the second round of MAKER were blasted against the NCBI non-redundant protein sequences database (NR) with blastp (-evalue 1e-6 -max_hsps 1 -max_target_seqs 1 -outfmt 6) as implemented in blast+ v2.6.0. All putative gene functions based on the best homology matches were annotated in the genome with a custom bash script (add_blast_annotation_custom.bash). Protein-coding genes were also searched for protein domains and signatures and annotated for InterPro (IPR), Pfam, and Gene Ontology (GO) terms using InterProScan v5.50-84.0 (Jones et al., 2014) and MAKER ipr_update_gff. Protein domains were exported as features in a GFF file using MAKER iprscan2gff3.

Finally, low-quality genes were identified with AGAT v0.6.0 (Dainat, 2021). These genes were filtered out if they were shorter than 50 amino acids and flagged if they had an incomplete open reading frame (ORF). Gene models produced by the second MAKER round were kept as the final reference dataset based on their higher number, AED distribution, and BUSCO completeness (Table S1 in File S2). Genome annotation was also inspected visually with JBrowse v1.1.10 (Skinner et al., 2009).

### General bioinformatics tools

After each assembly, scaffolds were sorted by size using seqkit v0.10.1 command sort -l -r -2 and renamed with command replace -p .+ -r "{nr}" (i.e. scaffold “1” being the longest, etc.). All alignment files were systematically sorted by leftmost coordinates, converted to BAM, and indexed with SAMtools v1.9. Alignment summary reports were produced with BAMtools v2.5.1 (Barnett et al., 2011). FASTQ files were converted in FASTA when needed with seqtk v1.3 (https://github.com/lh3/seqtk), and similarly, GFFs were converted to GTF with AGAT v0.6.0. Analyses were performed on Rāpoi, the Victoria University of Wellington high-performance computer cluster. Analyses requiring R scripts were performed in R v4.02 (R Core Team, 2020) on RStudio (RStudio Team, 2020).

## References

Aljanabi, S. M., & Martinez, I. (1997). Universal and rapid salt-extraction of high quality genomic DNA for PCR-based techniques. *Nucleic Acids Research*, *25*(22), 4692–4693. https://doi.org/10.1093/nar/25.22.4692

Andrews, S. (2018). *FastQC: A quality control tool for high through-put sequence data*. http://www.bioinformatics.babraham.ac.uk/projects/fastqc

Austin, C. M., Tan, M. H., Harrisson, K. A., Lee, Y. P., Croft, L. J., Sunnucks, P., Pavlova, A., & Gan, H. M. (2017). De novo genome assembly and annotation of Australia’s largest freshwater fish, the Murray cod (*Maccullochella peelii*), from Illumina and Nanopore sequencing read. *GigaScience*, *6*(8), 1–6. https://doi.org/10.1093/gigascience/gix063

Bao, W., Kojima, K. K., & Kohany, O. (2015). Repbase Update, a database of repetitive elements in eukaryotic genomes. *Mobile DNA*, *6*(1), 11. https://doi.org/10.1186/s13100-015-0041-9

Barnett, D. W., Garrison, E. K., Quinlan, A. R., Stromberg, M. P., & Marth, G. T. (2011). BamTools: a C++ API and toolkit for analyzing and managing BAM files. *Bioinformatics*, *27*(12), 1691–1692. https://doi.org/10.1093/bioinformatics/btr174

Broad Institute. (2019). *Picard toolkit*. Broad Institute, GitHub Repository. http://broadinstitute.github.io/picard/

Bushnell, B. (2018). *BBMap short read aligner*. Berkeley: University of California. http://sourceforge.net/projects/bbmap

Camacho, C., Coulouris, G., Avagyan, V., Ma, N., Papadopoulos, J., Bealer, K., & Madden, T. L. (2009). BLAST+: architecture and applications. *BMC Bioinformatics*, *10*(1), 1–9. https://doi.org/10.1186/1471-2105-10-421

Challis, R. (2017). *rjchallis/assembly-stats 17.02*. Zenodo. https://doi.org/https://doi.org/10.5281/zenodo.322347

Chen, Y., Wan, S., Li, Q., Dong, X., Diao, J., Liao, Q., Wang, G.-Y., & Gao, Z.-X. (2021). Genome-Wide Integrated Analysis revealed functions of lncRNA–miRNA–mRNA interaction in growth of intermuscular bones in *Megalobrama amblycephala*. *Frontiers in Cell and Developmental Biology*, *8*(603815), 1–15. https://doi.org/10.3389/fcell.2020.603815

Cheng, H., Concepcion, G. T., Feng, X., Zhang, H., & Li, H. (2021). Haplotype-resolved de novo assembly using phased assembly graphs with hifiasm. *Nature Methods*, *18*(2), 170–175. https://doi.org/10.1038/s41592-020-01056-5

Dainat, J. (2021). *AGAT: Another Gff Analysis Toolkit to handle annotations in any GTF/GFF format. (Version v0.6.0)*. Zenodo. https://doi.org/https://www.doi.org/10.5281/zenodo.3552717

Danecek, P., Auton, A., Abecasis, G., Albers, C. A., Banks, E., DePristo, M. A., Handsaker, R. E., Lunter, G., Marth, G. T., Sherry, S. T., McVean, G., & Durbin, R. (2011). The variant call format and VCFtools. *Bioinformatics*, *27*(15), 2156–2158. https://doi.org/10.1093/bioinformatics/btr330

De Coster, W. (2017). *Per base sequence content and quality (new basecaller)*. https://gigabaseorgigabyte.wordpress.com/2017/05/10/per-base-sequence-content-and-quality-new-basecaller/

De Coster, W., D’Hert, S., Schultz, D. T., Cruts, M., & Van Broeckhoven, C. (2018). NanoPack: visualizing and processing long-read sequencing data. *Bioinformatics*, *34*(15), 2666–2669. https://doi.org/10.1093/bioinformatics/bty149

Eddy, S. R. (2011). Accelerated Profile HMM Searches. *PLoS Computational Biology*, *7*(10), e1002195. https://doi.org/10.1371/journal.pcbi.1002195

Feron, R., Zahm, M., Cabau, C., Klopp, C., Roques, C., Bouchez, O., Eché, C., Valière, S., Donnadieu, C., Haffray, P., Bestin, A., Morvezen, R., Acloque, H., Euclide, P. T., Wen, M., Jouano, E., Schartl, M., Postlethwait, J. H., Schraidt, C., … Guiguen, Y. (2020). Characterization of a Y-specific duplication/insertion of the anti-Mullerian hormone type II receptor gene based on a chromosome-scale genome assembly of yellow perch, *Perca flavescens*. *Molecular Ecology Resources*, *20*(2), 531–543. https://doi.org/10.1111/1755-0998.13133

Flynn, J. M., Hubley, R., Goubert, C., Rosen, J., Clark, A. G., Feschotte, C., & Smit, A. F. (2020). RepeatModeler2 for automated genomic discovery of transposable element families. *Proceedings of the National Academy of Sciences*, *117*(17), 9451–9457. https://doi.org/10.1073/pnas.1921046117

Gan, W., Chung-Davidson, Y. W., Chen, Z., Song, S., Cui, W., He, W., Zhang, Q., Li, W., Li, M., & Ren, J. (2021). Global tissue transcriptomic analysis to improve genome annotation and unravel skin pigmentation in goldfish. *Scientific Reports*, *11*(1), 1–14. https://doi.org/10.1038/s41598-020-80168-6

Holt, C., & Yandell, M. (2011). MAKER2: an annotation pipeline and genome-database management tool for second-generation genome projects. *BMC Bioinformatics*, *12*(491), 1–14. https://doi.org/10.1186/1471-2105-12-491

Iwasaki, W., Fukunaga, T., Isagozawa, R., Yamada, K., Maeda, Y., Satoh, T. P., Sado, T., Mabuchi, K., Takeshima, H., Miya, M., & Nishida, M. (2013). MitoFish and MitoAnnotator: A mitochondrial genome database of fish with an accurate and automatic annotation pipeline. *Molecular Biology and Evolution*, *30*(11), 2531–2540. https://doi.org/10.1093/molbev/mst141

Jain, C., Koren, S., Dilthey, A., Phillippy, A. M., & Aluru, S. (2018). A fast adaptive algorithm for computing whole-genome homology maps. *Bioinformatics*, *34*(17), i748–i756. https://doi.org/10.1093/bioinformatics/bty597

Jansen, H. J., Liem, M., Jong-Raadsen, S. A., Dufour, S., Weltzien, F.-A., Swinkels, W., Koelewijn, A., Palstra, A. P., Pelster, B., Spaink, H. P., Thillart, G. E. van den, Dirks, R. P., & Henkel, C. V. (2017). Rapid de novo assembly of the European eel genome from nanopore sequencing reads. *Scientific Reports*, *7*(1), 7213. https://doi.org/10.1038/s41598-017-07650-6

Jiang, J. B., Quattrini, A. M., Francis, W. R., Ryan, J. F., Rodríguez, E., & McFadden, C. S. (2019). A hybrid de novo assembly of the sea pansy (*Renilla muelleri*) genome. *GigaScience*, *8*(4), 1–7. https://doi.org/10.1093/gigascience/giz026

Jones, P., Binns, D., Chang, H.-Y., Fraser, M., Li, W., McAnulla, C., McWilliam, H., Maslen, J., Mitchell, A., Nuka, G., Pesseat, S., Quinn, A. F., Sangrador-Vegas, A., Scheremetjew, M., Yong, S.-Y., Lopez, R., & Hunter, S. (2014). InterProScan 5: genome-scale protein function classification. *Bioinformatics*, *30*(9), 1236–1240. https://doi.org/10.1093/bioinformatics/btu031

Kearse, M., Moir, R., Wilson, A., Stones-Havas, S., Cheung, M., Sturrock, S., Buxton, S., Cooper, A., Markowitz, S., Duran, C., Thierer, T., Ashton, B., Meintjes, P., & Drummond, A. (2012). Geneious Basic: An integrated and extendable desktop software platform for the organization and analysis of sequence data. *Bioinformatics*, *28*(12), 1647–1649. https://doi.org/10.1093/bioinformatics/bts199

Keel, B. N., & Snelling, W. M. (2018). Comparison of Burrows-Wheeler transform-based mapping algorithms used in high-throughput whole-genome sequencing: Application to illumina data for livestock genomes 1. *Frontiers in Genetics*, *9*(35), 1–6. https://doi.org/10.3389/fgene.2018.00035

Kersey, P. J., Allen, J. E., Armean, I., Boddu, S., Bolt, B. J., Carvalho-Silva, D., Christensen, M., Davis, P., Falin, L. J., Grabmueller, C., Humphrey, J., Kerhornou, A., Khobova, J., Aranganathan, N. K., Langridge, N., Lowy, E., McDowall, M. D., Maheswari, U., Nuhn, M., … Staines, D. M. (2016). Ensembl Genomes 2016: more genomes, more complexity. *Nucleic Acids Research*, *44*(D1), D574–D580. https://doi.org/10.1093/nar/gkv1209

Kolmogorov, M., Yuan, J., Lin, Y., & Pevzner, P. A. (2019). Assembly of long, error-prone reads using repeat graphs. *Nature Biotechnology*, *37*(5), 540–546. https://doi.org/10.1038/s41587-019-0072-8

Korf, I. (2004). Gene finding in novel genomes. *BMC Bioinformatics*, *5*(59). https://doi.org/10.1186/1471-2105-5-59

Li, H. (2011). A statistical framework for SNP calling, mutation discovery, association mapping and population genetical parameter estimation from sequencing data. *Bioinformatics*, *27*(21), 2987–2993. https://doi.org/10.1093/bioinformatics/btr509

Li, H. (2013). *Aligning sequence reads, clone sequences and assembly contigs with BWA-MEM*. https://arxiv.org/abs/1303.3997v2

Li, H. (2018). Minimap2: pairwise alignment for nucleotide sequences. *Bioinformatics*, *34*(18), 3094–3100. https://doi.org/10.1093/bioinformatics/bty191

Li, H., & Durbin, R. (2009). Fast and accurate short read alignment with Burrows-Wheeler transform. *Bioinformatics*, *25*(14), 1754–1760. https://doi.org/10.1093/bioinformatics/btp324

Li, H., Handsaker, B., Wysoker, A., Fennell, T., Ruan, J., Homer, N., Marth, G., Abecasis, G., & Durbin, R. (2009). The Sequence Alignment/Map format and SAMtools. *Bioinformatics*, *25*(16), 2078–2079. https://doi.org/10.1093/bioinformatics/btp352

Marçais, G., & Kingsford, C. (2011). A fast, lock-free approach for efficient parallel counting of occurrences of *k*-mers. *Bioinformatics*, *27*(6), 764–770. https://doi.org/10.1093/bioinformatics/btr011

Miller, J. R., Delcher, A. L., Koren, S., Venter, E., Walenz, B. P., Brownley, A., Johnson, J., Li, K., Mobarry, C., & Sutton, G. (2008). Aggressive assembly of pyrosequencing reads with mates. *Bioinformatics*, *24*(24), 2818–2824. https://doi.org/10.1093/bioinformatics/btn548

Nudelman, G., Frasca, A., Kent, B., Sadler, K. C., Sealfon, S. C., Walsh, M. J., & Zaslavsky, E. (2018). High resolution annotation of zebrafish transcriptome using long-read sequencing. *Genome Research*, *28*(9), 1415–1425. https://doi.org/10.1101/gr.223586.117

Nurk, S., Walenz, B. P., Rhie, A., Vollger, M. R., Logsdon, G. A., Grothe, R., Miga, K. H., Eichler, E. E., Phillippy, A. M., & Koren, S. (2020). HiCanu: Accurate assembly of segmental duplications, satellites, and allelic variants from high-fidelity long reads. *Genome Research*, *30*(9), 1291–1305. https://doi.org/10.1101/GR.263566.120

PacBio. (2020). *SMRT Link v9.0*. https://www.pacb.com/support/software-downloads/

Piccoli, G. R. (2021). *grpiccoli/assemblies-stats: (Version 1.1.1)*. Zenodo. https://doi.org/10.5281/zenodo.4703697

Quinlan, A. R., & Hall, I. M. (2010). BEDTools: a flexible suite of utilities for comparing genomic features. *Bioinformatics*, *26*(6), 841–842. https://doi.org/10.1093/bioinformatics/btq033

R Core Team. (2020). *R: A language and environment for statistical computing*. R Foundation for Statistical Computing. http://www.r-project.org/

Roach, M. J., Schmidt, S. A., & Borneman, A. R. (2018). Purge Haplotigs: allelic contig reassignment for third-gen diploid genome assemblies. *BMC Bioinformatics*, *19*(460), 1–10. https://doi.org/10.1186/s12859-018-2485-7

RStudio Team. (2020). *RStudio: Integrated development environment for R*. RStudio, PBC. http://www.rstudio.com/

Shen, W., Le, S., Li, Y., & Hu, F. (2016). SeqKit: A cross-platform and ultrafast toolkit for FASTA/Q file manipulation. *PLOS ONE*, *11*(10), e0163962. https://doi.org/10.1371/journal.pone.0163962

Simão, F. A., Waterhouse, R. M., Ioannidis, P., Kriventseva, E. V., & Zdobnov, E. M. (2015). BUSCO: assessing genome assembly and annotation completeness with single-copy orthologs. *Bioinformatics*, *31*(19), 3210–3212. https://doi.org/10.1093/bioinformatics/btv351

Skinner, M. E., Uzilov, A. V., Stein, L. D., Mungall, C. J., & Holmes, I. H. (2009). JBrowse: A next-generation genome browser. *Genome Research*, *19*(9), 1630–1638. https://doi.org/10.1101/gr.094607.109

Smit, A., Hubley, R., & Green, P. (2013). *RepeatMasker Open-4.0*. http://www.repeatmasker.org

Stanke, M., Steinkamp, R., Waack, S., & Morgenstern, B. (2004). AUGUSTUS: a web server for gene finding in eukaryotes. *Nucleic Acids Research*, *32*(Web Server), W309–W312. https://doi.org/10.1093/nar/gkh379

Storer, J., Hubley, R., Rosen, J., Wheeler, T. J., & Smit, A. F. (2021). The Dfam community resource of transposable element families, sequence models, and genome annotations. *Mobile DNA*, *12*(2), 1–14. https://doi.org/10.1186/s13100-020-00230-y

Tan, M. H., Austin, C. M., Hammer, M. P., Lee, Y. P., Croft, L. J., & Gan, H. M. (2018). Finding Nemo: hybrid assembly with Oxford Nanopore and Illumina reads greatly improves the clownfish (*Amphiprion ocellaris*) genome assembly. *GigaScience*, *7*(3), 1–6. https://doi.org/10.1093/gigascience/gix137

Thai, B. T., Lee, Y. P., Gan, H. M., Austin, C. M., Croft, L. J., Trieu, T. A., & Tan, M. H. (2019). Whole genome assembly of the snout otter clam, *Lutraria rhynchaena*, using Nanopore and Illumina data, benchmarked against bivalve genome assemblies. *Frontiers in Genetics*, *10*(1158), 1–8. https://doi.org/10.3389/fgene.2019.01158

Trincado, J. L., Entizne, J. C., Hysenaj, G., Singh, B., Skalic, M., Elliott, D. J., & Eyras, E. (2018). SUPPA2: Fast, accurate, and uncertainty-aware differential splicing analysis across multiple conditions. *Genome Biology*, *19*(1), 1–11. https://doi.org/10.1186/s13059-018-1417-1

Vezzi, F., Narzisi, G., & Mishra, B. (2012a). Reevaluating assembly evaluations with Feature Response Curves: GAGE and assemblathons. *PLoS ONE*, *7*(12), e52210. https://doi.org/10.1371/journal.pone.0052210

Vezzi, F., Narzisi, G., & Mishra, B. (2012b). Feature-by-Feature – Evaluating De Novo Sequence Assembly. *PLoS ONE*, *7*(2), e31002. https://doi.org/10.1371/journal.pone.0031002

Vurture, G. W., Sedlazeck, F. J., Nattestad, M., Underwood, C. J., Fang, H., Gurtowski, J., & Schatz, M. C. (2017). GenomeScope: fast reference-free genome profiling from short reads. *Bioinformatics*, *33*(14), 2202–2204. https://doi.org/10.1093/bioinformatics/btx153

Wen, M., Ng, J. H. J., Zhu, F., Chionh, Y. T., Chia, W. N., Mendenhall, I. H., Lee, B. P.-H., Irving, A. T., & Wang, L.-F. (2018). Exploring the genome and transcriptome of the cave nectar bat Eonycteris spelaea with PacBio long-read sequencing. *GigaScience*, *7*(10), 1–8. https://doi.org/10.1093/gigascience/giy116

Wood, D. E. (2019). *MiniKraken2 v2 8GB database*. Johns Hopkins University. ftp://ftp.ccb.jhu.edu/pub/data/kraken2_dbs/old/minikraken2_v2_8GB_201904.tgz

Wood, D. E., Lu, J., & Langmead, B. (2019). Improved metagenomic analysis with Kraken 2. *Genome Biology*, *20*(257), 1–13. https://doi.org/10.1186/s13059-019-1891-0

Zhang, X., Li, G., Jiang, H., Li, L., Ma, J., Li, H., & Chen, J. (2019). Full-length transcriptome analysis of *Litopenaeus vannamei* reveals transcript variants involved in the innate immune system. *Fish & Shellfish Immunology*, *87*, 346–359. https://doi.org/10.1016/j.fsi.2019.01.023

Zimin, A. V., Marçais, G., Puiu, D., Roberts, M., Salzberg, S. L., & Yorke, J. A. (2013). The MaSuRCA genome assembler. *Bioinformatics*, *29*(21), 2669–2677. https://doi.org/10.1093/bioinformatics/btt476

Zimin, A. V., Puiu, D., Luo, M.-C., Zhu, T., Koren, S., Marçais, G., Yorke, J. A., Dvořák, J., & Salzberg, S. L. (2017). Hybrid assembly of the large and highly repetitive genome of *Aegilops tauschii*, a progenitor of bread wheat, with the MaSuRCA mega-reads algorithm. *Genome Research*, *27*(5), 787–792. https://doi.org/10.1101/gr.213405.116

Zimin, A. V., & Salzberg, S. L. (2020). The genome polishing tool POLCA makes fast and accurate corrections in genome assemblies. *PLoS Computational Biology*, *16*(6), 1–8. https://doi.org/10.1371/journal.pcbi.1007981
