# Supplementary material for: Genome assembly and isoform analysis of a highly heterozygous New Zealand fisheries species, the tarakihi (Nemadactylus macropterus)
Source: G3 (Bethesda). 2022 Dec 8;13(2):jkac315. doi: 10.1093/g3journal/jkac315 (PMC9911067; doi:10.1093/g3journal/jkac315)
Supplement: jkac315_Supplementary_Data [file jkac315_supplementary_data.zip › File_S2_G3-2022-403723.docx]

# **Supplementary Material S2**

# Genome assembly and isoform analysis of a highly heterozygous New Zealand fisheries species, the tarakihi (*Nemadactylus macropterus*)

Yvan Papa, Maren Wellenreuther, Mark A. Morrison, Peter A. Ritchie

## Supplementary Figures


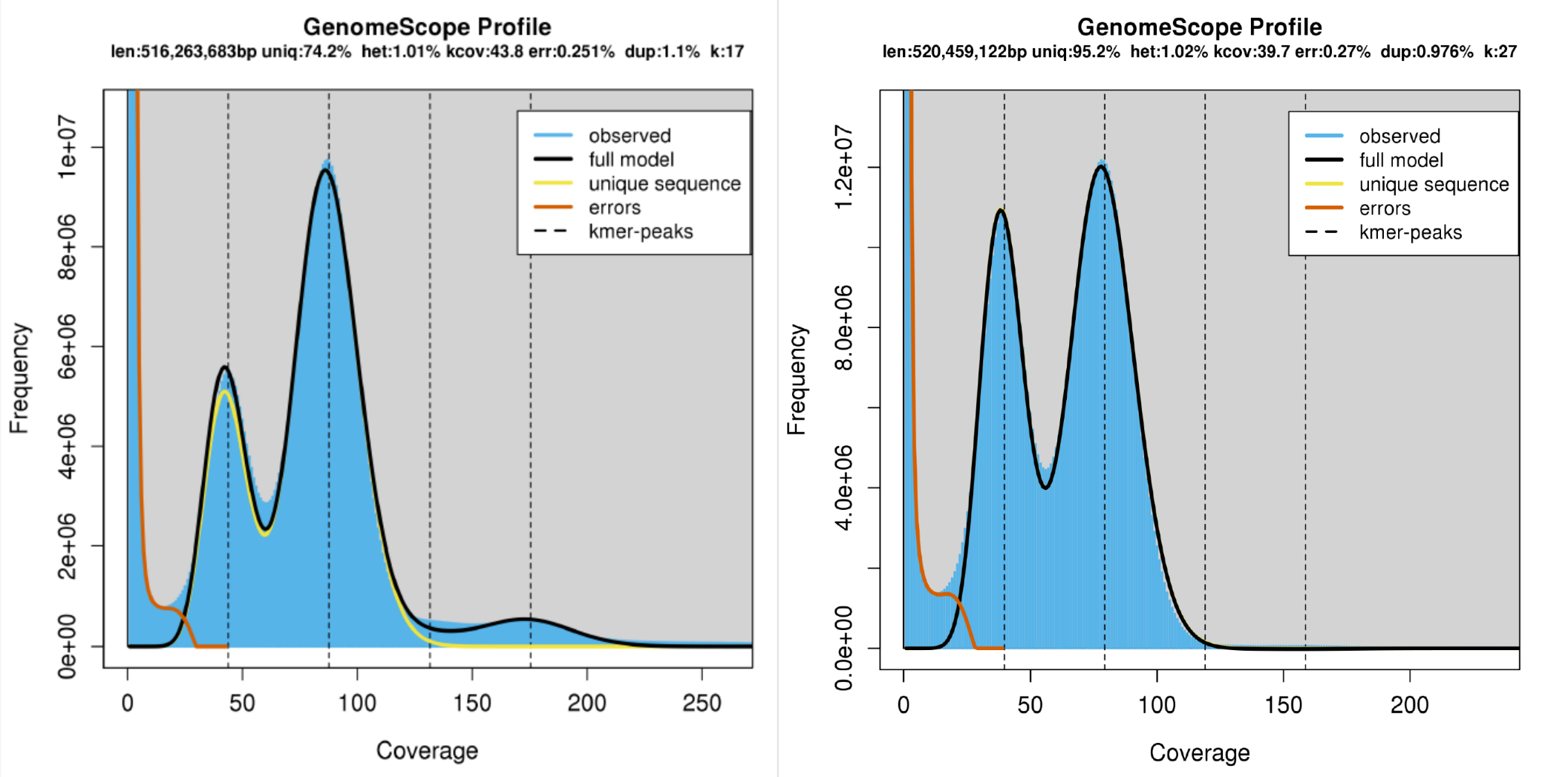


Figure S1. Histograms of 17- and 27-mer frequency in clean Illumina reads. Estimation of genome size of tarakihi, heterozygosity, and duplicated regions. The first and second peaks show the k-mer frequency of heterozygous and homologous regions, respectively.


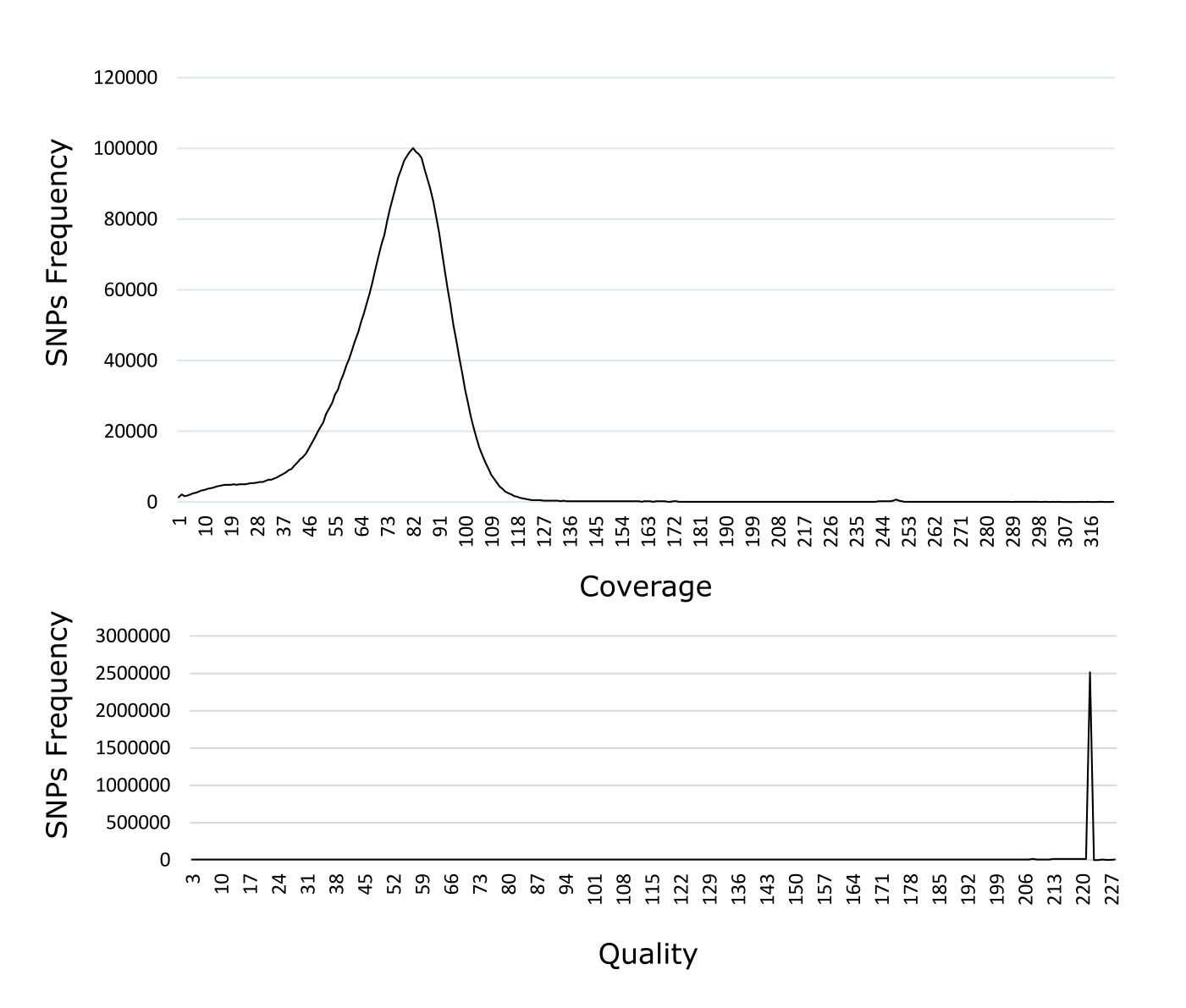


Figure S2. Distribution of coverage (top) and quality (bottom) of SNPs called from Illumina reads back to the tarakihi assembly. SNPs were filtered for a minimum genotype depth of 20 according to the increase in steepness starting approximately at this point. Quality was always high, so the default site quality value was used.


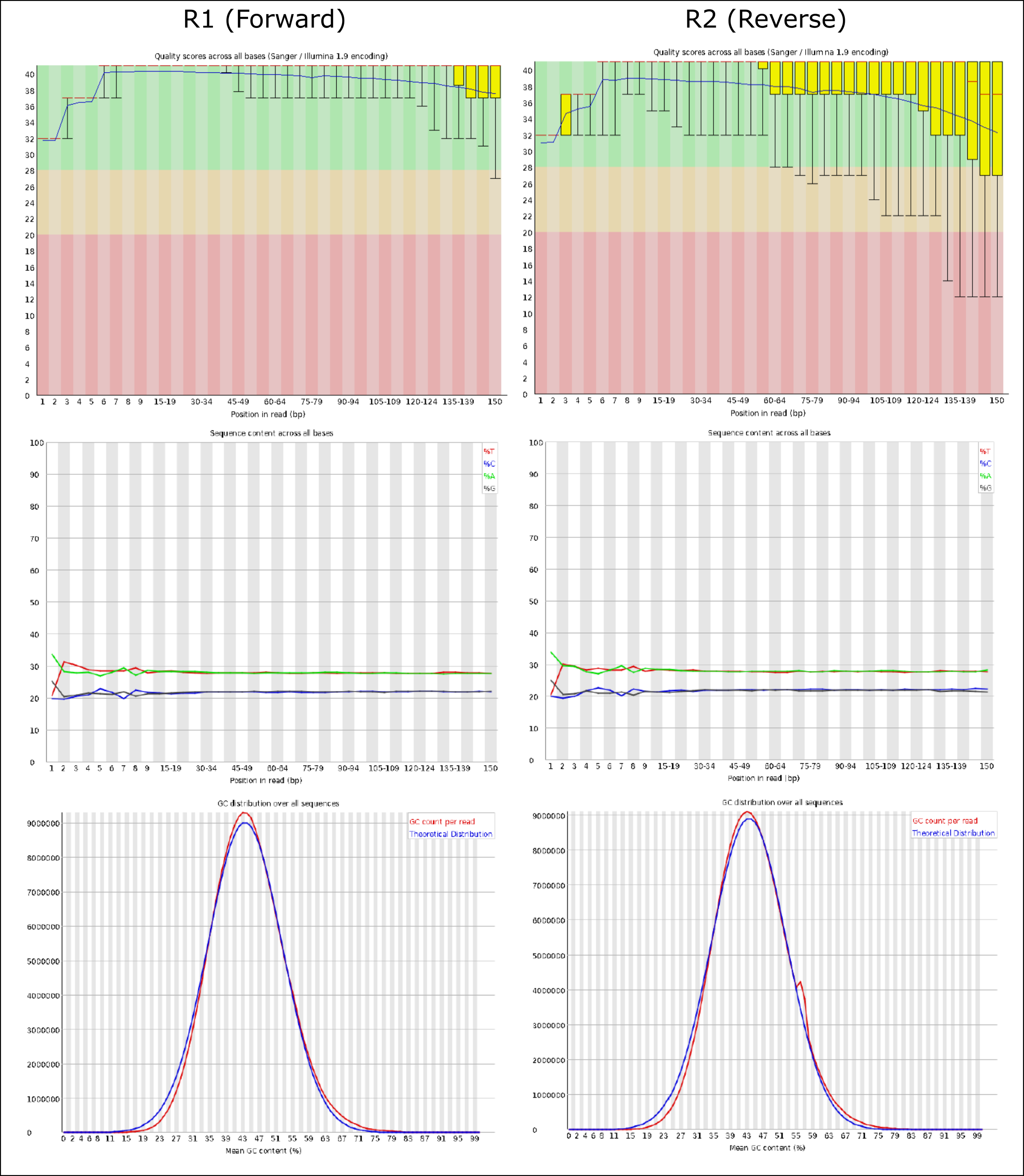


Figure S3. Some of FastQC quality metrics results, for forward (R1) and reverse (R2) sequencing reads of tarakihi. Top: Per base sequence quality. Middle: Per base sequence base content. Bottom: GC distribution over all sequences. See main text for the explanation on the slight bias in bases content for the first few bases in all reads. GC content of reverse reads detected a few over-represented sequences, which were most probably harmless sequencing artifacts that should be discarded during the quality control step of the MaSuRCA assembly.


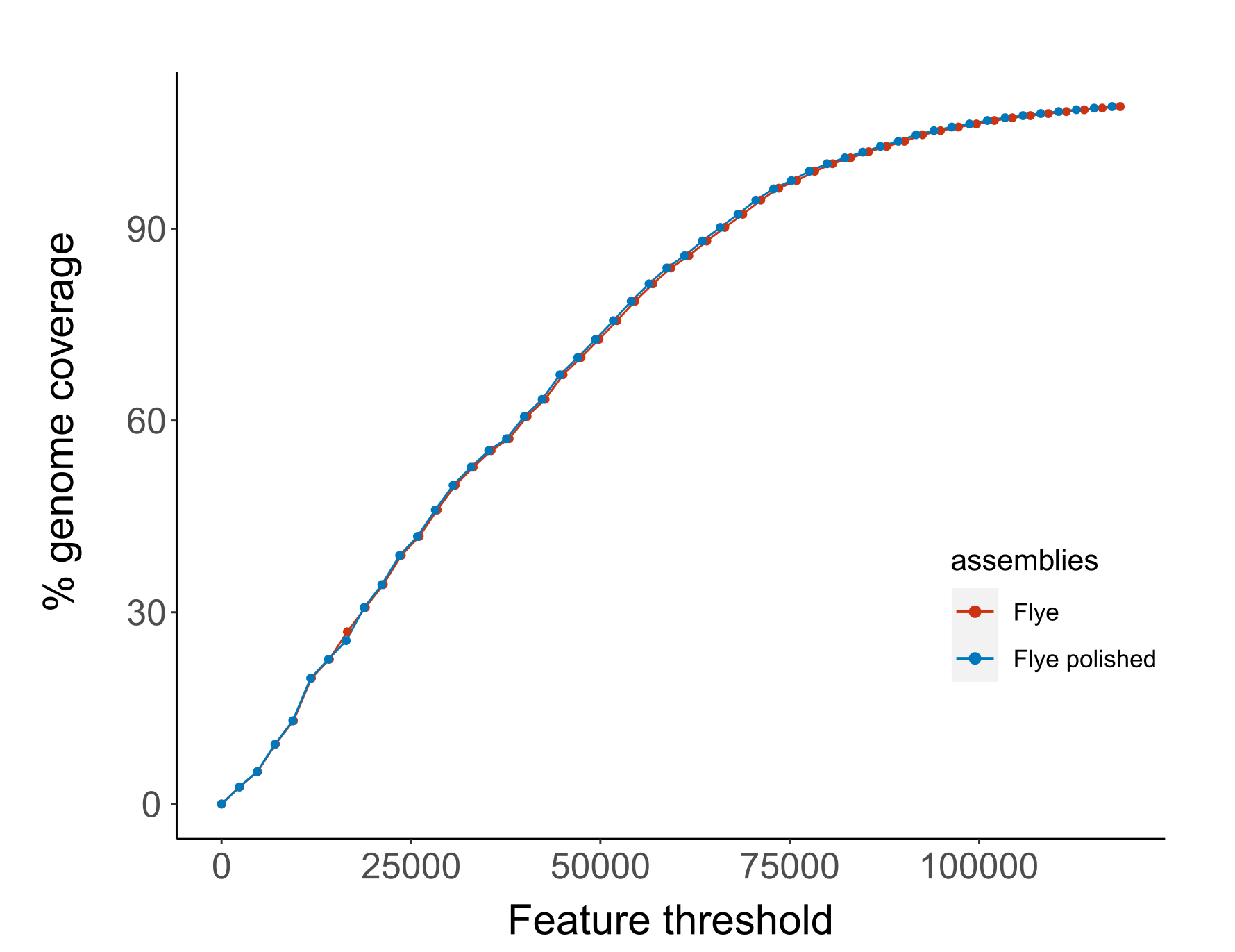


Figure S4. FRC curves as shown in Figure 3, but with only the Flye and Flye polished assemblies projected for better visualization. The Y-axis represents the cumulative size of the assembly and the X-axis is the cumulative number of potential errors (i.e. “features”). Assemblies for which the curves are steeper are considered more accurate. For the same cumulative genome size, the Flye unpolished assembly always accumulated slightly more potential errors (i.e. features).


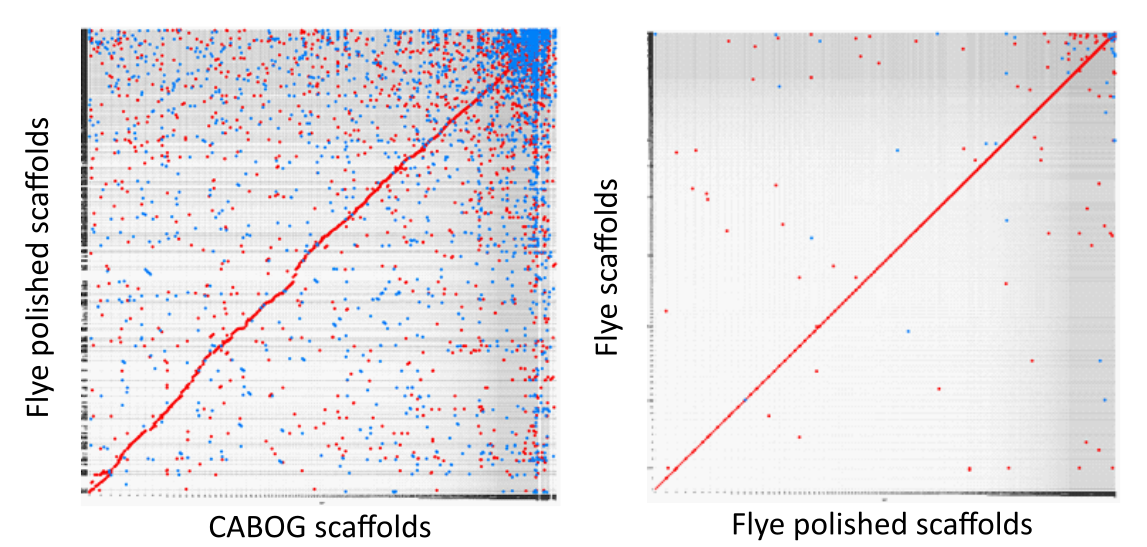


Figure S5. Plots of pairwise alignment scores between scaffolds of the tarakihi assemblies, obtained with MashMap. Each dot represents a match between the query and the reference sequence. Colors correspond to the strand direction (red for positive, blue for negative).


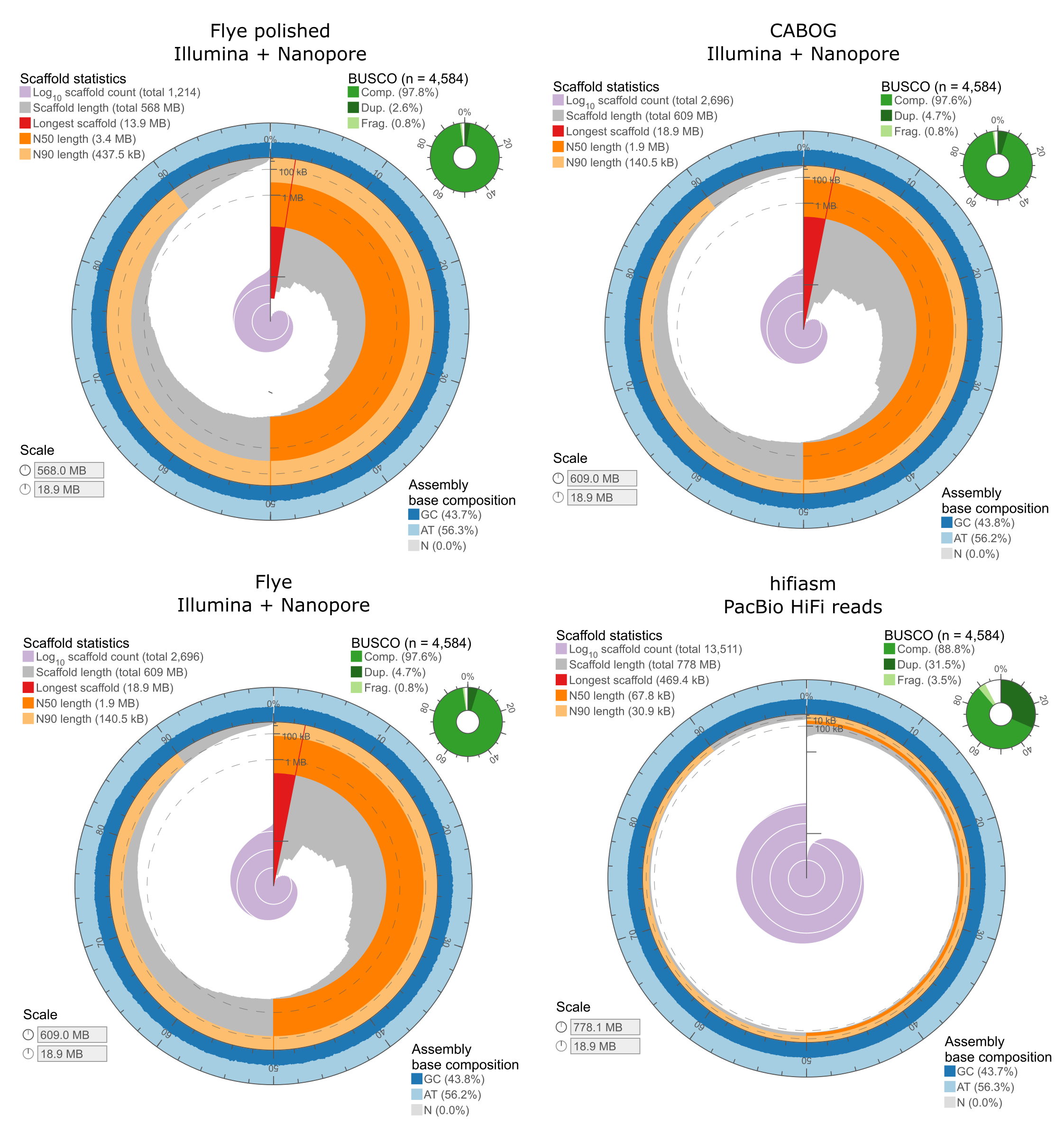


Figure S6. Visualization of contiguity and completeness of the four tarakihi assemblies produced.

## Supplementary Tables

Table S1. Quality control statistics of the gene models obtained after different rounds of MAKER.

|  | Round 1 | Round 2 | Round 3 | |
| --- | --- | --- | --- | --- |
| Number of gene models | 9,008 | 20,327 | 19,930 |  |
| Average gene length | 11,455 | 13,741 | 14,057 |  |
| AED ≤ 0.5 | 100% | 95.50% | 94.00% |  |
| Complete BUSCO transcripts | 58.70% | 76.00% | 74.90% |  |

Table S2. Main classes and proportions of repeat elements detected in the tarakihi genome.

| Repeat type | No. of elements | Length occupied (bp) | % in the genome |
| --- | --- | --- | --- |
| Retroelements | 323634 | 35060271 | 6.17 |
| SINEs | 33606 | 2627490 | 0.46 |
| Penelope | 8128 | 793327 | 0.14 |
| LINEs | 214886 | 24389420 | 4.29 |
| CRE/SLACS | 1 | 69 | 0 |
| L2/CR1/Rex | 139371 | 15942708 | 2.81 |
| R1/LOA/Jockey | 6466 | 805273 | 0.14 |
| R2/R4/NeSL | 5543 | 659291 | 0.12 |
| RTE/Bov-B | 24021 | 2686662 | 0.47 |
| L1/CIN4 | 12218 | 1572014 | 0.28 |
| LTR elements | 75142 | 8043361 | 1.42 |
| BEL/Pao | 6293 | 705505 | 0.12 |
| Ty1/Copia | 3175 | 392807 | 0.07 |
| Gypsy/DIRS1 | 36293 | 4302376 | 0.76 |
| Retroviral | 15032 | 1103666 | 0.19 |
| DNA transposons | 578638 | 61749831 | 10.87 |
| hobo-Activator | 293706 | 32369155 | 5.7 |
| Tc1-IS630-Pogo | 80564 | 7567833 | 1.33 |
| En-Spm | 0 | 0 | 0 |
| MuDR-IS905 | 0 | 0 | 0 |
| PiggyBac | 13600 | 1089280 | 0.19 |
| Tourist/Harbinger | 44201 | 5208743 | 0.92 |
| Other | 11367 | 1081984 | 0.19 |
| Rolling-circles | 35706 | 2925989 | 0.52 |
| Unclassified | 458433 | 60021928 | 10.57 |
| Total interspersed repeats |  | 156832030 | 27.62 |
| Small RNA | 9364 | 715919 | 0.13 |
| Satellites | 6240 | 816959 | 0.14 |
| Simple repeats | 238583 | 10392751 | 1.83 |
| Low complexity | 28658 | 1630008 | 0.29 |
